# Collective Cell Motion in an Epithelial Sheet Can Be Quantitatively Described by a Stochastic Interacting Particle Model

**DOI:** 10.1371/journal.pcbi.1002944

**Published:** 2013-03-07

**Authors:** Néstor Sepúlveda, Laurence Petitjean, Olivier Cochet, Erwan Grasland-Mongrain, Pascal Silberzan, Vincent Hakim

**Affiliations:** 1 Laboratoire de Physique Statistique, CNRS, Université P et M Curie, Université Paris Diderot, Ecole Normale Supérieure, Paris, France; 2 Institut Jean Le Rond D'Alembert, UMR 7190 CNRS-UPMC, Paris, France; 3 Fondation Pierre Gilles de Gennes pour la Recherche, Paris, France; 4 Laboratoire Physico-chimie Curie, Institut Curie, CNRS, Université Pierre et Marie Curie, Paris, France; Harvard University, United States of America.

## Abstract

Modelling the displacement of thousands of cells that move in a collective way is required for the simulation and the theoretical analysis of various biological processes. Here, we tackle this question in the controlled setting where the motion of Madin-Darby Canine Kidney (MDCK) cells in a confluent epithelium is triggered by the unmasking of free surface. We develop a simple model in which cells are described as point particles with a dynamic based on the two premises that, first, cells move in a stochastic manner and, second, tend to adapt their motion to that of their neighbors. Detailed comparison to experimental data show that the model provides a quantitatively accurate description of cell motion in the epithelium bulk at early times. In addition, inclusion of model “leader” cells with modified characteristics, accounts for the digitated shape of the interface which develops over the subsequent hours, providing that leader cells invade free surface more easily than other cells and coordinate their motion with their followers. The previously-described progression of the epithelium border is reproduced by the model and quantitatively explained.

## Author Summary

Living organisms, from bacteria to large mammals, move not only as single entities but also in groups. This is true for cells in multicellular organisms. The group or collective motion of cells is an important component of development as well as processes like cancer and wound healing. To better understand this phenomenon, we have recorded the displacement of cells as they move collectively on a substrate and invade free space. The results can be accurately described by modelling the motion of cells as random but with a tendency to move at the same velocity as their neighbors. This allows us to analyze conditions under which the invasion of free space takes place, guided by a few cells that have become different of the others, as observed in the experiments. The developed model should serve as a useful basis for the description of other processes that involve collective cell motion.

## Introduction

Interactions between moving entities correlates their motions. This takes place at all scales, from atoms and molecules, as evidenced by the familiar experiences of wind and fluid vortices to the astronomical scales of stars and galaxies. In the biological realm, collective movements are observed from colonies of bacteria [1] to herds of animals [2]. They underlie the fascinating motions of bird flocks [3], [4] and fish schools [5] as well as pedestrian track patterns and traffic flows [6]. In these different cases, the motion of the individual organism is very complex and difficult to describe in a detailed way. However, simple models that captures important features of the interaction have proven useful for the description of collective movements. For instance, that car drivers reduces their speed when car density increases is a key property for traffic jam formation. At the level of cells, collective motion is an important component of different biological processes in multicellular organisms [7]. It is an integral part of development [8], as illustrated for instance by dorsal closure in *Drosophila* embryo, maintenance processes such as wound healing [9], and disorders with cancer as a prime example [10]. It has been studied *in vivo*, in model systems such as border cell migration in drosophila oogenesis [11], [12] or lateral line migration in zebrafish [13], [14], as well as in simpler and more controlled *ex vivo* experiments where the motion of cells is simpler to record [15]–[21].

Many aspects of the migratory behavior of cells in two dimensions have thus been studied by using the classical “wound healing” scratch assay, in which a confluent epithelium is scratched with a tool such as a pipette cone or a razor blade, so as to mechanically remove a “strip of cells” from the monolayer. The progression of the remaining cells during the “healing” of this “wound” is then observed under the microscope for up to a few days. In previous works [17], [19], [22], we developed and studied a very reproducible version of these experiments in which a portion of the culture plate is masked by microfabricated stencils. Stencils removal unmasks surfaces free of cells. This produces well-defined “wounds” with rectilinear edges and precisely controlled widths and it triggers cell movements. In the subsequent hours, cells invade the free surface under the apparent guidance of “leader” cells [15], [17], [22].

Our understanding of the mechanisms that coordinate the behavior of multiple cells in these different processes is far from complete. A model of collective cell motion should be useful to try and precisely describe these diverse phenomena. It should also allow to test and quantify the effect of different perturbations [18], [23]. A pioneeringly simple description of the collective behavior of self-propelled particles in general has been proposed by Vicsek et al [24] based on interacting and stochastically moving particles. Several authors have since carefully analyzed this model [25]–[27] as well as related ones [28]–[30]. Coordinated motion of active cells has been modelled along this line [31]–[34] or with more extended cell descriptions [35], [36] as well as with continuum descriptions [37]–[39]. While these previous models provide insights in coordinated cell motion, continuum models do not account for the stochastic character of individual cell motion and simplifying assumptions, such as the use of discrete time and/or velocities of fixed-modulus, in other models [24], [25], [31], [40], prevent detailed comparisons to experimental data. Thus, our aim here is to obtain a minimal model that quantitatively describes coordinated cell motion. We compare the developed model to motion of cells recorded in our experiments, as obtained from Particle-Image Velocimetry (PIV). We find, using numerical simulations that the model accounts quite precisely for the collective cell movements studied at early times before the appearance of leader cells. We further incorporate fast-moving leader cells and determine the conditions under which they guide collective motion as in the experiments.

## Results

### Model of collective cell motion

Our aim is to describe in the simplest quantitative fashion the collective motion of cells in an actively moving epithelium. We wish in particular to take into account that cells are actively motile [16]–[18], that the motion of a cell is stochastic [16], [41], [42] and that it is influenced by interactions with its neighbors. We also wish to obtain a model of minimal computational complexity that can provide a description of a large population of moving cells. A particle-based model appears best suited to this task. We thus propose and study a model of stochastically moving objects biased by their interactions with their neighbors. Each cell is reduced to its center point, the dynamics of which is described by a Langevin-like equation in continuous-time. The velocity 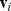 of the cell 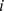 is a real two-dimensional vector which evolves as,
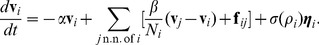
(1)where the summation on the right-hand-side (r. h. s.) is performed on the 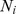 cells 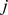 that are the nearest neighbors of cell 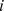. Thus, the behavior of a cell in our model is only influenced by by its closest neighbors, that is cells that are supposed to be in direct contact with it. We describe here the model main characteristics (see *Methods* for implementation details). We consider cells that are actively motile and explore their environment in a random fashion [16], [41], [42]. This is modelled in a classical way by a noisy drive (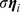), here described as an Ornstein-Uhlenbeck process with correlation time 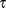. The noise amplitude is first taken to be a constant, . It is then generalized to a decreasing function of the local cell density 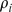 to describe the dependence of the mean cell speed on cell density. The linear damping term (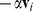) is meant to account in an effective way for dissipative processes coming from rupture of adhesive contacts or friction with the substrate or other cells. Finally, the motion of cell 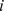 is influenced by its interaction with a neighbor in two ways. First, its velocity tends to become equal to the velocity of the neighboring cell 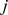 [24], [25] with a strength determined by the coupling constant 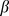. Second, the fact that cells do not overlap and have a maximal extent is taken into account by the force 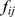 between neighbor cells 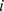 and 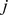, which is repulsive with a hard-core at short distances and attractive at longer distances [40], [43]. These interactions are sketched in Figure S1.

### The model provides an accurate description of experimental data at early times

**Figure 1:**
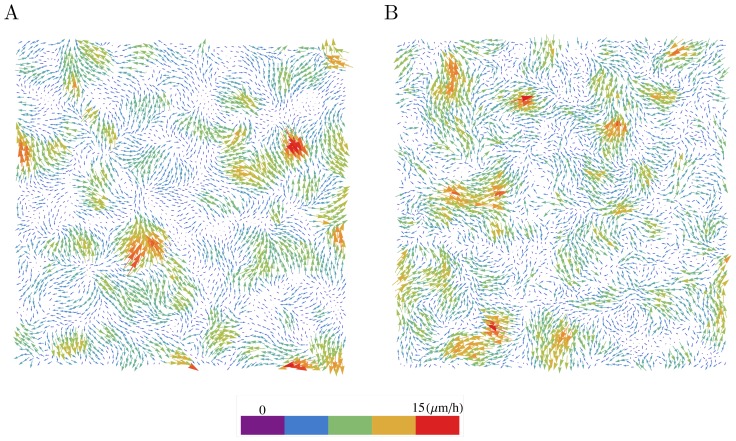
Cell velocity fields at early times. The cell velocity field is displayed 30 min after removal of the stencil. (A) Experiment (B) Numerical simulation of the model. Box size 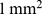. The parameters of the simulated model are 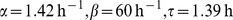 and 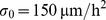 (constant amplitude noise).

**Figure 2:**
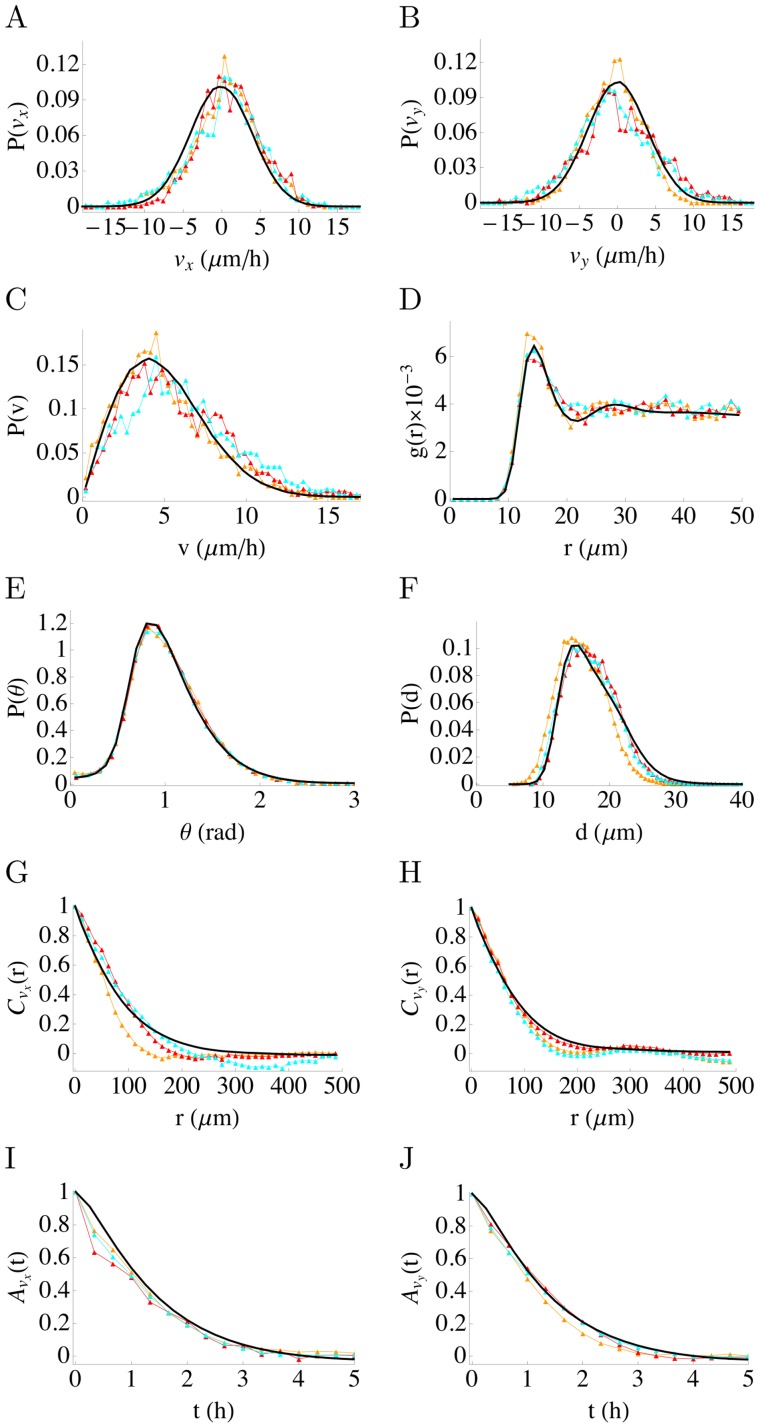
Statistical characterizations of the cell velocity field and positions at early time. Different statistical quantities are computed for the velocity fields (30 min after stencil removal) in the experiments and model. Results of numerical simulations of the model are shown as solid black curves (the simulation parameter values are the same as in Figure 1). The experimental data of 3 different experiments are shown as colored symbols. (A) and (B) Distribution of the components 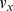 and 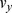 of the cell velocities, (C) Distribution of the cell velocity moduli, (D) Spatial correlation of the cell centers, (E) Distribution of angle between two successive neighbors of a cell (see *Methods*), (F) Distribution of distance between the centers of two neighboring cells, (G) and (H) spatial velocity correlation for the components 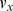 and 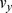 of the velocity respectively, (I) and (J) temporal velocity correlation for the components 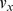 and 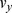 of the velocity respectively.

Stencil removal in the experiments rapidly increases cell motility in the whole epithelium. Complex displacement fields are observed that can precisely be measured by PIV analysis as described in previous works [17], [19] and shown in Figure 1A. The histogram of the velocity component normal to the epithelium border 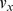 is identical to the histogram of 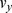, the velocity component parallel to the epithelium border as seen in Figure 2 A,B, showing that cell motion is isotropic at early times. After a couple of hours, leader cells appear and guide cell invasion of the free surface. Cell motion then becomes dissymmetric along the 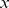 and 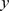 axes.

The model with a constant noise amplitude, 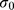, was simulated with a number of model cells (N = 4000) comparable to the number of cells in the experiments. Its parameters were adjusted to the experimental data at early time (30 min after stencil removal) by fitting correlation functions computed from model simulations to experimental velocity fields provided from PIV analysis, as described in *Methods*. With the obtained parameters, the simulated and experimental velocity field appeared very similar as can be seen in Figure 1. This was quantitatively assessed by comparing different statistical quantities for the model and experimental data. The 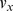 and 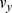 histograms closely match and are both well described by the same gaussian (Figure 2 A, B and Figure S2 A, B which displays the data plotted in log-linear coordinates, to better show the histogram tails). Similarly, the distribution of the velocity moduli is close to the corresponding Maxwellian distribution (Figure 2 C and and Figure S2 C). The experimental equal-time spatial velocity correlation (Figure 2 G, H and Figure S2 D, E) as well as the velocity field auto-correlation (Figure 2 I, J and Figure S2 F, G) are both well fitted by the model. The similarity of the correlation of the 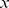 and 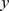 velocity components in these plots makes further apparent the isotropy of the cell dynamics at early times. The velocity correlation length is remarkably long of the order of 150 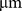 or about ten cells, as noted previously [17], [19], [21]. The cell velocity auto-correlation decays with a time scale of about one hour which quantifies the time during which cells maintain their velocity. This time scale is comparable to the characteristic organization time of microtubules [44] and to the 50 min that we previously measured [22] for the reorientation of the microtubule organizing center relative to the nucleus.

The model provides a good description of the statistical structure of the cell velocity field, while correctly accounting for the spatial relations between neighboring cells (Figure 2 D, E, F). It reproduces the correlation function of cell positions, that is the probability density of finding a cell center at a distance 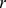 of another cell (Figure 2 D) as well as the distribution of distances between neighboring cells (Figure 2 F). Finally, taking the center of a cell as origin, the rotation angle between the positions of two of it successive neighbors is shown in Figure 2 E. The average angle is 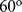 as it should, and the whole angle distribution is seen to be very similar for the real and model cells (Figure 2 E). In summary, we find that the interacting particle model described by Eq. [1] with a constant noise amplitude succeeds in capturing quite precisely cell dynamics in the epithelium bulk. As shown in Figure S3, the model continues to describe well the distribution and correlation of the cell velocity component 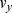 parallel to the band border in the epithelium bulk for a few hours. However as time passes, the border motion influences more and more the motion of cells in the epithelium bulk as shown by the progressive departure of 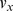 distributions and correlations from their initial values (see Figure S3).

In spite of the model simplicity, it is difficult to obtain exact expressions for the statistical quantities displayed in Figure 2. In order to better understand the influence of the different parameters, we considered the analytically solvable approximation of the model obtained by computing the time evolution of cell velocities as given by Eq. [1], but with the cell positions fixed at the vertices of a triangular lattice, as described in *Text S1*. The obtained expressions for the distribution of cell velocities and for the velocity correlation functions approximate that of the full model and describe their dependence on different parameters. As shown in *Text S1*, the noise amplitude 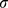 determines the cell speed scale but does not influence the shape of the cell speed distribution or the normalized velocity correlation function. Figure S4 illustrates the influence of the parameters 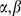 and 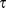 on the velocity correlation functions. An increase of 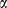 diminishes both the spatial and temporal extent of the velocity correlations. An increase of 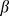 increases correlation spatially but has almost no effect on temporal correlations. On the contrary, an increase of 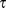 increases correlations in time but has a very weak influence on their spatial extent.

In the above-described experiments, cell density does not strongly vary in the epithelium bulk at early time. The mean cell speed however depends on the cell density [45] as shown by its decrease as cells reach confluence before stencil removal (see Figure 1A in ref. [19]), as well as by the observed correlation between cell speed and cell density in migrating bands that exhibit large density heterogeneities. In order to account for this effect, we generalized the model to include a dependence of the noise amplitude 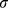 on the local cell density (see *Methods*), as written in Eq. [1], since 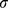 is the main model parameter that controls the mean cell speed. As shown in Figure S5, the inclusion of this dependence does not significantly change the agreement between model and experimental data at early times. It plays however an important role in the epithelium motion at later times, as described below.

### Leader cells and fingers at the epithelium border

**Figure 3:**
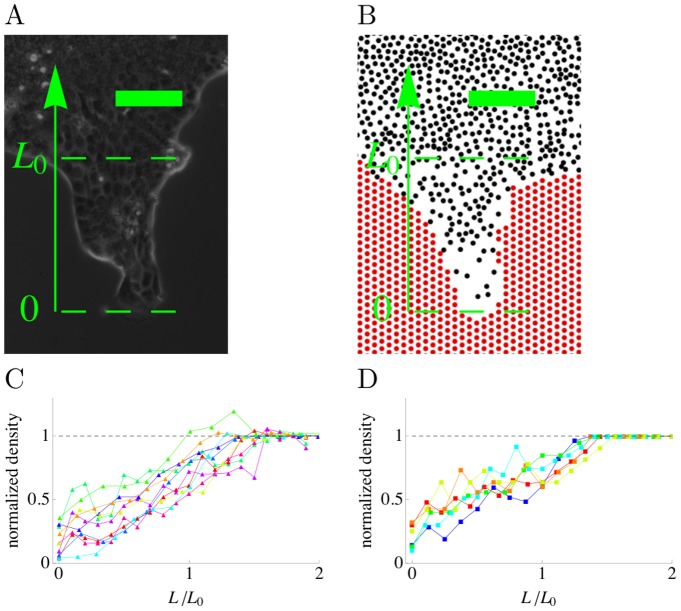
Finger shapes. Experiments (left : A, C) and simulations (right : B, D). In the simulated model, the noise amplitude depends on the density (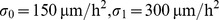 see *Methods*). Other parameters are the same as in Figure 1. (A) and (B) Examples of finger shapes. (C) and (D) Cell density in fingers as a function of the position along the finger (scale bar : 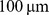).

Since the proposed model described well the coordinated motion of cells at early times, we investigated whether it could also reproduce the behavior of the epithelium during the whole duration of the experiments. After a couple of hours, leader cells appear and are observed to guide MDCK cell motion at the epithelium border in the form of “fingers” that invade free space, as described in previous works [15], [17], [22] and shown in Figure 3. Different suggestions have previously been made as to the origin of fingers and leader cells. Proposed mechanisms include diffusion and chemo-attraction [37], [39] as well as an intrinsic instability [46] of the cell border hypothesized to be driven by an increased border speed in its outward curving parts [47]. Leader cells are about three times larger than their followers, with a size of the order of 50 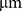 as compared to 15–25 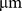 for other cells. They also move faster than other cells, do not divide and are often binucleated (see Figure S6). Thus, we here take the more conservative viewpoint that leader cells have acquired different characteristics from other cells in the epithelium. We study whether the observed fingers and border movement can be reproduced by introducing a few modified cells in our model.

The dynamics of leader cells differ in several ways from those of other cells. The velocities of leader cells are found to be constant in modulus and direction to a good approximation (see Figure 7 in ref. [17]) with velocity moduli peaked around 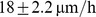. Contrary to other cells, leader cells display a very active lamellipodium along their whole membrane in contact with the free surface. They actively invade free surface while other cells do not. This difference in explorative behaviors may stem from the actin cable that follows the epithelium border [48], [49] and is only interrupted in leader cells. Finally, the motion of a leader cell is not independent of that of other cells. Cutting a leader cell from the following cells strongly perturbs its dynamics. Its motion becomes erratic and it regains its characteristics only after re-adhesion to the epithelium [22].

We introduced model leaders cell in our simulations that took into account these different properties in a simple way (see *Methods* for details). Leader cells were created as faster cells at the epithelium border with a fixed outward predetermined velocity. The difference in explorative behaviors between leader and other cells was accounted for, in an effective manner, by a repellent force felt by non-leader cells at the epithelium border upon exploration of free surface. This repulsion disappeared as soon as the surface had been explored by a leader or another non-leader cell (see *Methods* for details). In addition, a leader cell was assumed to coordinate its motion with the cells directly following it. Namely, a leader cell was assumed to slow down when it was too fast for its followers.

Fingers produced with these prescriptions resemble those observed in experiments as shown in Figure 3 A, B and in Figure S7. In the observed experimental fingers, the cell density is lower than in the epithelium bulk. It decreases continuously from the finger bases to their leader cell tip, as quantified previously [22] (Figure 3 C). A very similar trend is observed in the fingers produced in the model as shown in Figure 3 D. The agreement between the experimental and model finger densities is actually surprisingly close given that the model only accounts for the increased spreading on the surface of fast moving cells but does not explicitly include more specific facts such as the more elongated shapes of cells in the fingers.

The effects of the different assumed properties was assessed by relaxing or modifying some of them. Feedback on the leader cell from its followers was found necessary to prevent detachment of the leader cell from the epithelium bulk. Narrow finger-like protrusions on the interface were only present when differences in explorative behaviors between leader cells and other cells were taken into account. Similarly, the noise amplitude dependence on local cell density provided a mean for follower cells to autonomously reach a high speed. In the model with a constant noise amplitude, the leader cell was observed to slow down. It adapted to the “unrestrained” border progression speed that follower cells would adopt in absence of free surface repulsion which is of the same order as cell speed in the bulk (i.e. about 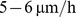). The increase of cell speed with decreasing cell density greatly increased the unrestrained border progression speed, as shown in Figure S8. This allowed leader cells to maintain a high speed when coordinating their motion with follower cells. A high border progression speed could in principle come from other mechanisms. We noted for instance that with a density-independent noise term, it could arise from the addition of cell division to the model which created an internal pressure in the epithelium. In the experiments, this alternative mechanism is probably not dominant since it is opposed by the fact that, in the epithelium bulk at high density, dividing cells have smaller areas and undergo contact inhibition [45].

### Motion of the epithelium border

Having obtained a quantitative model of cell motion in the epithelium bulk and of cell entrainment by a leader cell, we investigated whether the appearance of a number of leader cells would be sufficient to account for the motion of the whole epithelium border. The rate of appearance of leader cells was measured in time and space along the epithelium border, in the experiments. It was found to be approximately constant in time and uniform along the border with a value of 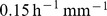 in the first 20 hours after stencil removal and a lower value of 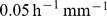 at later times (see Figure S9). It was also approximately uniform in space except that the probability of a leader cell appearance within a lateral distance of 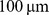 of another leader cell was found to be very low.

Simulations of the cell model were thus performed with the measured rate of leader cell creation (see *Methods*).The direction of leader cell velocities was taken normal to the initial epithelium border. The moduli of their velocities were drawn according to a gaussian distribution with parameters determined by the measured leader cell velocity distribution. With a fixed number of cells, the epithelium border speed initially increased. However, the border progression then slowed down and eventually stopped when cells reached their maximal size (given in the model by the attractive part of the cell-cell interaction potential). Cell division was thus incorporated in the model. In order to avoid creating spurious internal pressure in the epithelium, a potential division was implemented only when it did not increase the cell density above the initial one (see *Methods*).

**Figure 4:**
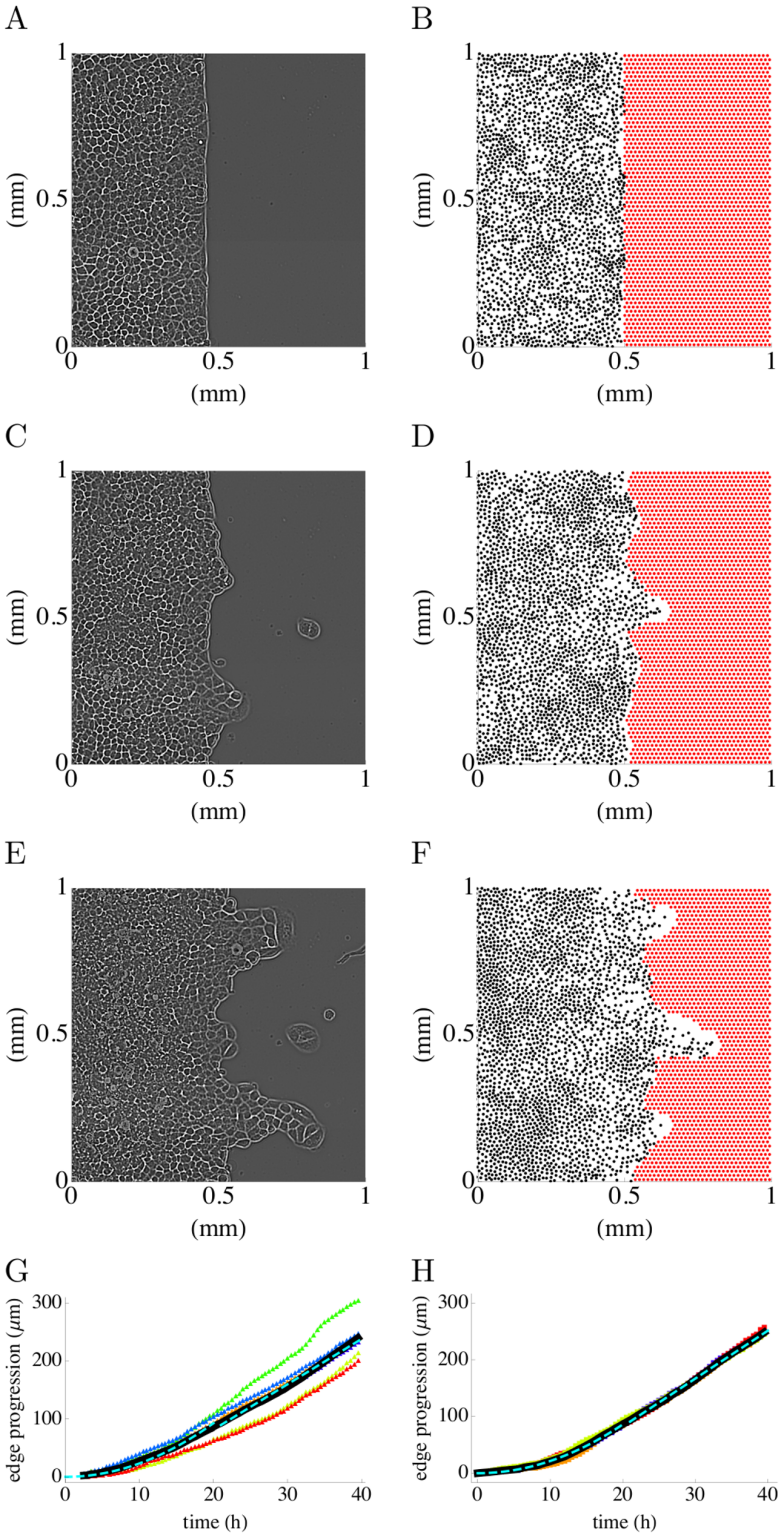
Border progression. Experiments (left column: A,C,E,G) and numerical simulations (right column : B,D,F,H). The model parameters are the same as in Figure 3. Pictures of the epithelium at 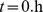 ((A), (B)), 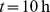 ((C),(D)) and 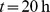 ((E),(F)). In the experimental pictures (A,C,E) the cells are shown. For the simulation, the positions of the particles are shown as black dots. (G) mean border progression in (n = 6) experiments shown as colored triangles; the average progression is shown as a solid black line together with a quadratic fit at early times (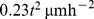) and a linear fit at late times (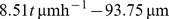)(dashed light blue lines) (H) mean border progression in (n = 6) simulations shown as colored squares; the average progression is shown as a solid black line together with a quadratic fit at early times (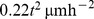) and a linear fit at late times (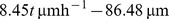) (dashed light blue lines). In the simulations, cell division was implemented, as described in *Methods*.

As shown in Figure 4, the simulated border shapes and movements closely resemble the experimentally observed ones (see also Video S1 and Video S2). The mean border progression also quantitively agrees with the experimentally measured ones as shown in Figure 4. Both display an early regime in which the mean border position grows as 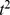 where 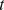 is the time elapsed since the unmasking of the free surface, as reported previously [17]. This is followed by a later regime in which the epithelium border mean position moves approximately linearly in time i.e. at a constant speed. The previous results and model actually provide a simple explanation of both regimes. Without leader cells, the epithelium border invades the free surface at a low speed. Each new leader cell that appears entrains at its higher progression speed a portion of the border the lateral extent of which is of the order of the velocity correlation length. Therefore, the mean speed of the border progression increases with the appearance of each new leader. This speed increase is linear in time for a constant rate a leader cell appearance, resulting in the 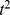 time progression of the border (see *Text S1* for mathematical details). Crossover to the second regime takes place when the leader cell creation rate becomes low and the number of fingers increases much more slowly (see Figure S9).

**Figure 5:**
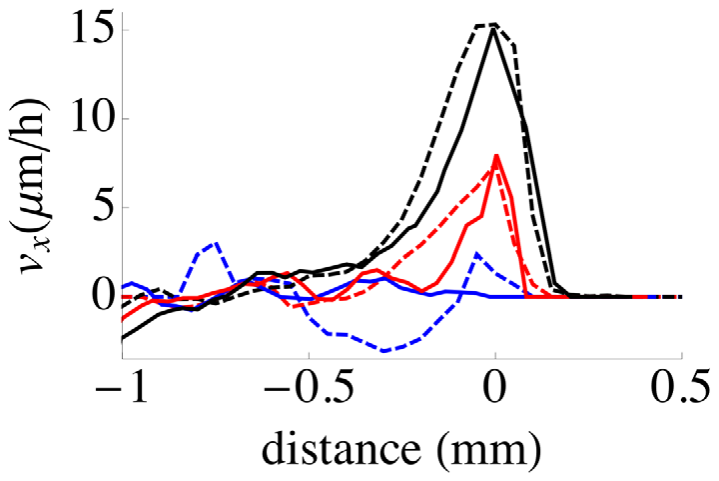
Average cell velocity profile around the epithelium border. The average component of the cell velocity (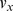) normal to the epithelium border in experiments (dashed lines) and simulations (solid lines) is plotted as a function of the distance to the mean position of the border at different times after free surface unmasking : 0 h (blue lines), 10 h (red lines) and 20 h (dark lines) (the line shown corresponds to average of 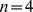 data sets (bands with 1.5 mm initial interface length were used in experiments). The average was performed as follows: for a given simulation or experiment, the mean border position was computed at the above times. At each of these times, the data of different experiments or simulations were first translated along the 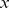-axis such that the mean border position coincided was positioned at 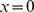. The average over different experiment was then performed (spatial bin size 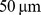.

Finally, we computed the mean component 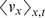 of the cell velocity normal to free border, at different times 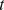 and at different distances 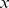 from the mean border position. As shown in Figure 5, the experimental and model velocity profiles 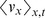 change as time evolves but both sets are very similar both in amplitude and scale over the whole time course of the experiment.

In summary, the developed model of cell motion in the epithelium bulk reproduces well the epithelium motion over the whole time course of our experiments, upon addition of leader cells with suitable properties.

## Discussion

Collective motion is a remarkable feature of the dynamics of different organisms, and simple models that appear to capture the essence of this phenomenon have attracted a lot of interest [24], [26]. Data collection is actively pursued for various types of coordinated movements (e. g. [3]) but detailed comparisons between models and experiments [2], [50] are still relatively scarce. We have developed a simple model to try and describe collective cell motion in an epithelium. The quantitative agreement between simulations and the experimental data demonstrates that our description based on the stochastic motion of interacting particules is indeed able to accurately capture the coordinated movement of cells. This agrees with a previously made analogy between cell motion in an epithelium and the dynamics of a complex fluid [21]. We have furthermore shown that adding to these interacting cells, model leader cells with suitable properties, reproduce the motion of the epithelium and of its border over the whole duration of our experiments. The results provide a simple explanation for the previously-reported different regimes of border progression at early and late times. The model should therefore prove helpful to better analyze the consequences of interactions between cells and of their perturbations in different contexts. It will hopefully also be of some use in more complex *in vivo* situations in which cell motion can be monitored [32], [51], [52].

Existing models of collective cell motion can be classified into three broad categories : model that include an extended description of the cell membrane, based for instance on a Potts model description [35], [36] or a vertex description [45], particle-based models [31]–[33] as the one here studied, and finally continuum descriptions [37]–[39] of tissue movement which do not explicitly describe individual cells. These three levels of description are complementary and each one has its own merits. Detailed cell descriptions allow for a more easy inclusion of biochemical and biophysical mechanisms while reduced ones are computationally more efficient, usually make use of less parameters and are easier to analyze mathematically. The model of interacting particles that we have analyzed in the present work, shares several general features with previously proposed models. The description is based as in refs. [31]–[33] on self-propelling particles that repel their neighbors when they are too close and attract them when they become more distant. The model includes a velocity alignment term as initially proposed in ref. [24]. However, to match the statistical properties of cell velocities in our experiment, the model departs from previous ones in significant ways. The motion of particles is not deterministic [33] but stochastic. Moreover, the model does not include a preferred cell speed and without added nonlinearity, a symmetry broken phase with spontaneously aligned velocities is precluded in our model. In this respect, it qualitatively differs from the family of models studied in ref. [24]–[27], [29]. It remains to be seen whether further nonlinearities will be needed to describe cell motion in different biological conditions. Some directions studied in extended cell models appear worth studying in extensions of the present model. For instance, inclusion of a cell area variable in our particle description would allow one to take advantage of the detailed model of cell division and contact inhibition proposed in [45]. Similarly, it has been found worth distinguishing cell velocities and cell polarization in extended cell models [35], [36]. Our model instead includes a memory of past velocities as in some single cell models [42]. A comparison of these two approaches should prove useful to the understanding of cell behavior in collective migration modes.

Several other features of cell motion in the proposed model deserve further attention. It should prove interesting to see how the model effective parameters emerge from more basic properties and, for instance, to elucidate whether the velocity alignment between a cell and its neighbors arises from adhesion, repeated encounters, signalling or a mix of these different processes. We have found that fingers comparable to experiments are produced when leader cells more actively invade free environment than following cells and also regulate their motion according to their contacts with following cells. The first property is reminiscent of the known role of leader cancer cells in three-dimensional geometry in degrading and remodelling the surrounding matrix to generate tracks for their followers [53], [54]. The second appears to accord with photo-ablation experiments which show that following cells provide important feedback for proper leader cell motion [22]. However, both properties need to be further investigated. We have introduced leader cells without specifying what induces a cell to become a leader. Determining the role in this transformation of chemical signalling and interface geometry and mechanics would allow one to relate the present model to previous proposals [37], [46]. The frequent appearance of bi-nucleated leader cells and the marked change of their creation rate after 20 hrs also point toward a role of the cell cycle that needs to be further investigated. Finally, experiments have started to classify on a large scale how different genes affect collective motion and to cluster them in different modules [18], [23], [55]. Further work is needed to see how these correlate with the few parameters of a simple model such as the one presented here.

## Methods

### Details of the model

The model describes a collection of 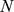 particles moving according to Eq. [1] of the main text. The noise term 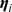 that drives the motion of cell 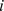 is taken to be an Ornstein-Uhlenbeck process with correlation time 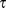


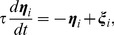
(2)with 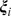 a delta-correlated white noise independently drawn in each cell (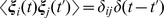). The force exerted by cell 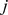 onto cell 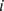 is taken under the form
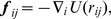
(3)with 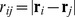 and the potential 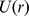 chosen as the sum of a repulsive short-range gaussian potential and an attractive part acting at longer distances,
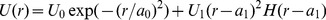
(4)with 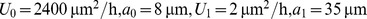, and 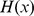 the Heaviside function 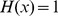 for 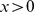 and 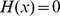 otherwise. In the simulations of cell motion in the epithelium bulk (Figure 1 and 2), the attractive part of the potential played no role and was omitted. In some exploratory simulations, the velocity alignment coupling 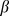 was chosen to be a decreasing function of the velocity difference between adjacent cells. However, this did not bring significant improvement to the fit between model and data and a constant coupling term was chosen, as described in Eq. [1].

Neighbors of a cell/particle needed to be defined to implement Eq. [1]. For computational efficiency, this was done as follows. The neighborhood of a particle 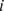 was split in 6 equal sectors (with the 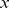-axis taken as one of the sector boundaries). The particle closest to particle 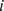 in each sector was taken to be a neighbor of particle 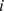 if its distance to particle 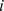 was less than 100 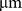. The local density at cell 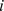 was computed as 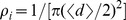, with 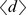 the average distance between a cell and its 6 neighbors (for sectors without neighbors the cut-off distance of 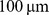 is taken). The distribution of angle between neighbors of a cell in Figure 2E was obtained by computing the absolute value of the angle between the vectors 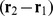 and 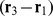 for each cell center position 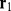, and every pair of center positions 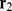 and 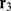 of its successive neighbors (i.e. neighbors of the chosen cell that are also themselves neighbors).

As described in the text and supplementary material, simulations were performed either with a constant noise amplitude 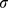 or with a noise amplitude that increased with decrease in local cell density 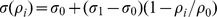 where 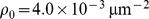 is the initial cell density in the band.

### Epithelium border, leader cells and cell interaction with free surface

After free surface unmasking, the epithelium border was straight and parallel to the y-coordinate axis. The epithelium border was defined in the simulations at later times, by taking the particle with the largest 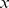-coordinate in successive bands of 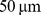 width in the 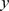 direction, covering the simulation space. Leader cells were introduced in the simulations, by randomly transforming into a leader cell a particle within 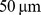 of the epithelium border and the y-coordinate of which was not within 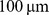 of an already created leader cell. The leader cells were created along the border length at an otherwise uniform rate in space and time of 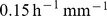 during the first 20 hours and 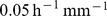 at later times. The leader cell velocity was chosen parallel to the 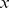-axis (i.e. normal to the epithelium initial border) with a modulus drawn according to a gaussian distribution of mean 18 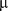
m/h with a standard deviation of 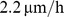. The velocity of a created leader cell was maintained constant at its initial velocity as long as it had 4 neighbors or more. Otherwise, it was given the mean velocity of its neighbors (with a maximum speed of 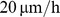) until the threshold number of 4 neighbors was reattained.

As described in the text, normal cell explore free surface less easily than leader cells. This was modelled by introducing a repulsive force exerted on normal cells upon invasion of unexplored surface, as follows. The unexplored surface was covered by 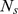 surface particles with a repulsive force 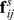 between pairs of particles (representing cells) and surface particles, closer than 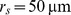. This force was added to the r. h. s. of Eq. [1] for the cell particles. The surface particles were assumed to disappear upon exploration of free surface by a particle associated to a leader or another cell. This was implemented as follows. A scalar ‘damage’ variable 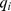 was associated to the surface particle 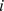. It was chosen to obey
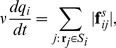
(5)with 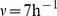. The summation on the r.h.s. of Eq. [5] was over the forces applied on the surface particle 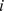 by the cells in its neighborhood 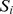, namely cells at a distance of the surface particle 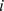 smaller than 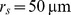. A surface particle was chosen to disappear when its damage variable reached the threshold value 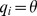 with 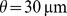.

The surface particle-normal cell interaction was modeled by
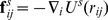
(6)where 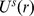 was taken to be of gaussian form 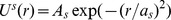 with values of parameters 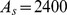 and 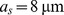. The same form was used for the force exerted by leader cells upon surface particles but with 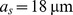 and a much larger amplitude 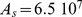 such that surface particles were quickly damaged by leader cells. Note that as the dynamics of leader cells was prescribed, there was no need to consider the force exerted by upon them by surface particles.

In the simulations with free-surface, cell division was implemented with a cell doubling time of 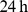. However, in order to avoid creating overpressure in the epithelium, a cell division was accepted only if it did not increase the local cell density 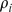 above the initial cell density 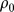. This resulted in modest increases in the number of cells 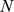 during the course of the simulations. For instance in the simulation displayed in Figure 4 B, D, F the number of cells was initially 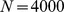, 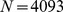 at 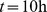 and finally, 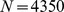 at 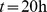.

### Simulations

Simulations were performed with custom computer codes. For parameter fitting and early time simulations, 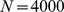 particles were used on a 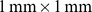 square with periodic boundary conditions, so that the cell density matched that of the experiments. For leader cells and border progression, 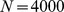 particles were used on a 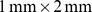 rectangle with periodic condition in the short 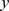-direction.

### Parameter fits

In order to determine the parameters that best fitted the experimental data at early time (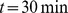 after mask removal; Figure 1 and 2), simulations served to compute, for given model parameters, both the equal time spatial velocity correlation function 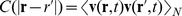 and the particle velocity autocorrelation 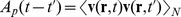 normalized by their value at 0 (as indicated by the subscript 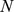). This was compared to the experimental data for the normalized cell equal-time velocity correlation 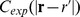 and for the normalized autocorrelation of the cell velocity field 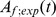 as provided by PIV (see below) by computing the mismatch 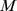.
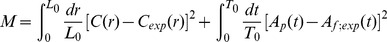
(7)with 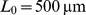 and 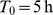 i.e. time and space intervals over which the experimental correlations significantly differed from zero. The minimization was performed with the Nelder-Mead simplex Amoeba algorithm [56]. The starting parameters were obtained from fitting in the same way the analytical expressions obtained for the model approximation with fixed particle positions (*SI text* section I). In this approximate model, noise amplitude does not influence the normalized correlations and it was determined from the mean cell speed after the determination of the other parameters. In the full model, the starting noise amplitude was taken equal to the one determined in the approximate model. It was recomputed to fit the mean cell speed after the determination of the other parameters by the Amoeba algorithm. The procedure was iterated until convergence. We compared the cell-velocity auto-correlation in the model to the auto-correlation of the velocity field in the experiment because the first is the most natural quantity for the model and the second a direct output of the PIV analysis of the experiment. In principle, these two quantities can differ since the first one corresponds to following a given cell and the second one to measure cell velocity at successive times at a fixed time of space (i.e. the first is a Lagrangian quantity whereas the second is an Eulerian one). To check that they were not significantly different in the present case, we computed the velocity field in the simulation by assigning to the center of each square of a space-covering grid (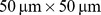 or 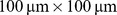, the mean velocity of particles in the considered square. As shown in Figure S10, for the two grids, the model velocity field auto-correlation was not found to be significantly different from the particle velocity auto-correlation. We also computed the cell velocity auto-correlation in the experiment by tracking individual cells after stencil removal. The obtained cell auto-correlation was also not found to significantly differ from the velocity field auto-correlation (Figure S11).

### Cell culture

MDCK cells [57] were cultured in Dulbecco's modified Eagle's medium supplemented with 10% FBS (Sigma), 2 mM L-glutamin solution (Gibco) and 1% antibiotic solution (penicillin (10,000 units/mL), streptomycin (10 mg/mL)). Cells were seeded and maintained at 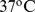, 5%CO2 and 90% humidity throughout the experiments.

### Microfabrication of the stencils and model wound

Microstencils were made of PDMS elastomer (Sylgard 184, Dow Corning) and prepared by classical microlithography as described elsewhere [17]. Experiments were performed in plastic six-well plates on the bottom of which microstencils were previously deposited. Cells were plated on the microstencils and cultured in the incubator until they reached confluence. At this time, the microstencils were peeled off.

### Time lapse microscopy

Time lapse acquisitions were performed on an automated inverted microscope (Olympus IX71) equipped with temperature, humidity and CO_2_ regulation. Displacements of the stage (Prior Scientific), and image acquisition (CCD camera (Retiga 400, QImaging), shutter (Uniblitz)) were computer-controlled through Metamorph (Universal Imaging). The delay between two successive frames was 5 min or 15 min. Experiments were performed for typically 40 hours.

### Image processing and velocity field measurement

The images were processed with ImageJ [Rasband W.S. 2007. ImageJ (National Institutes of Health, Bethesda), available at http://rsb.info.nih.gov/ij/] using a watershed plugin (available at http://bigwww.epfl.ch/sage/soft/watershed/index.html) to extract the contours when needed. The velocity field in the monolayer was mapped by PIV analysis using the MatPIV [58] software package version 1.4 for MatLab (MathWorks Inc.) [Matpiv is a GNU public license software (www.math.uio.no/jks/matpiv/)] as previously described in [19]. The data in Figure 1 and Figure 2 were obtained from squares of 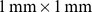 in the center of bands of cells of 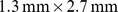.

## Supporting Information

Figure S1Sketch of the model. In the model each particle (here depicted by a small disk or a circle) has its own velocity (thin solid arrow). A given particle (denoted here by a solid black circle) interacts with its nearest neighbors (solid red disks). It moves under the action of three forces (large open arrows) which modify the particle velocity: one stochastic force 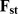 and two interaction forces, an alignment force between velocities 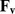 and a force generated by a potential 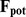. The chosen potential is such that 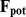 is repulsive at short distance (here represented by the dark grey disk), vanishes in an intermediate range of distances (white annulus) and is attractive in a still larger-range of distances (lighter annulus). Note that a particle interacts only with its nearest neighbors and not with particles that are farther from it (denoted here by open red circles).(TIF)

Figure S2 Statistical characterization of the cell velocity field, tails of the distribution functions. Data shown in Figure 2 of the main text are replotted in log-linear plots to better display the tails of the cell speed distribution functions and of the velocity-velocity correlations. The model results are depicted by solid black lines and the results of 3 experiments are depicted colored triangles and lines. (A, B, C) Probability distributions of the components of the velocity and of the velocity modulus. They are well-approximated respectively by Gaussian and Lorentzian distributions. (D,E) Equal time correlations of the velocity components as a function of distance. The experimental data show large relative fluctuations at long distances and the correlations become small. In this region, experimental data can take negative values and the corresponding colored lines are interrupted since the data cannot be plotted in log coordinate. (F,G) Temporal auto-correlations of the velocity components are close to exponential both in the model and in the experiments.
(TIF)

Figure S3Statistical characterization of the cell velocity as in Figure 2 of the main text but at later times after stencil removal: 1 h (A1, B1, C1, D1), 2 h (A2, B2, C2, D2) and 3 h (A3,B3,C3,D3). As in Figure 2, experimental data (colored symbols) are taken from the motions of cell in a center square of 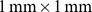 in 3 experiments with initial bands of cells of size 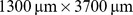. The solid lines show the corresponding model fits 30 min after stencil removal as in Figure 2. Panels (A) and (B) display 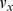 and 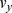 velocity component distributions. Panels (C) and (D) show correlations of 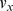 and 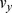 velocity components as a function of cell distances.The 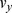 distributions and 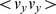 correlations remain in good agreement with the model fit. As time passes, the 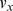 distributions and correlations depart from the early fits and from the corresponding 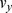 functions since border motion starts to influence cell motion in the center of the band.(TIF)

Figure S4Analytical approximations of correlation functions with cell centers fixed on a triangular lattice showing the dependence of the correlation functions on different parameters. Panels (A),(C),(E) show the normalized spatial velocity correlations as a function of distance and Panels (B),(D),(F) the normalized velocity autocorrelations as a function of time. The parameter 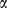 is varied in (A) and (B). The parameter 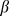 is varied in (C) and (D). The parameter 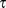 is varied in (E) and (F). The solid black lines are drawn for the values of 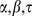 given in Figure 1 of the main manuscript, the dashed black lines for a two times larger value of the varied parameter and the dotted lines for a half as large value. Experimental data are shown by colored symbols for reference. One can note in (B),(D),(F) that the time velocity autocorrelation decays more slowly in the approximation that in the model with moving cells (compare with Figure 2 I in the main text).(TIF)

Figure S5Statistical characterizations of the cell velocity field and positions at early time (30 min after stencil removal). Same as Figure 1 and Figure 2 of the main text for a model with noise amplitude varying with density 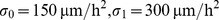 see *Methods*). Other parameters are the same as in Figure 1 and Figure 2 of the main text. Results of numerical simulations of the model are shown as solid black curves. The experimental data of 3 different experiments are shown as colored symbols for comparison. (A) Example of velocity field in the experiment and (B) in the simulation. (C) Distribution of the cell velocity moduli, (D) spatial correlation of the cell centers, (E) distribution of the angle between two successive neighbors of a cell (see text), (F) distribution of the distance between the centers of two neighboring cells, (G) and (H) spatial velocity correlation for the components 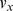 and 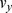 of the velocity respectively, (I) and (J) temporal velocity correlation for the components 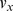 and 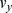 of the velocity respectively.(TIF)

Figure S6Picture of a leader cell and a finger. Cells were fixed and stained for nuclei with DAPI (blue) and for F-actin with alexa488-conjugated phalloidin (bar: 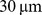). It is clearly seen that the leader cell is bi-nucleated and that it displays a well-developed lamellipodium.(TIF)

Figure S7 (A, B, C, D) Some simulated fingers with the free-surface repellent particles shown in red. The free-surface particles were used to draw the solid line which was considered as the finger contour. This contour was used to measure the finger density as a function of position, as shown in Figure 3 D of the manuscript.
(TIF)

Figure S8Border progression without the addition of leader cells and restrained by free -surface repulsion (left column A,C,E,G) or “unrestrained” i. e. without surface repulsion (right column, B,D,F,H). Images of two simulations at different times 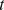 after free-surface unmasking (A & B, 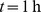; C & D, 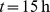; E & F, 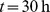). G & H: Border progression in (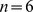) simulations (colored symbols) together with their average (solid black line) and linear fits (dashed light blue line which give a mean border progression speed of 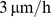 (G) in the restrained case and of 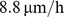 (H) in the unrestrained case.(TIF)

Figure S9 Experimental data on leader appearance along the epithelium border. (A) Total number of leader cells as a function of time in different experiments corresponding to a cumulated epithelium border length of 17 mm. (B) Same data showing the number of leader cells appearing during different time intervals (time bin : 5 h).
(TIF)

Figure S10Comparison of the velocity auto-correlation function for the particles and for the velocity field in the model with constant noise amplitude. The cell velocity autocorrelation is shown as a continuous curve. The velocity field was defined by attributing to the center of each square of a square-grid the mean velocity of the particles that it contained. Two different squares grids were used of step size 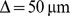 and 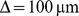 (dashed and dotted curves). The corresponding auto-correlation are shown as dashed (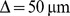) and dotted (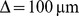) curves. The different curves are very close. To gauge the difference in parameter estimates, the model velocity field auto-correlations were used to fit the PIV velocity field. The obtained values of the parameters did not significantly differ from those used in Figure 1 and Figure 2 (the obtained 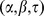 were (1.43, 38, 1.40) for the continuous curve, (1.42, 38,1.41) for the dashed curve; and (1.42, 38, 1.40) for the dotted curve).(TIF)

Figure S11Comparison of the cell velocity auto-correlation functions (circles, 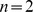 experiments) to velocity field auto correlation (triangles; 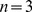 experiments) in the experiments. (A) Autocorrelation of the 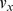 velocity component. (B) autocorrelation of the 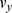 velocity component.(TIF)

Text S1 Velocity correlation functions in an analytically solvable approximation of the model and some simple estimates on border progression resulting from leader cell creation.
(PDF)

Video S1 Movie showing the cell motions and the epithelium border progression in one experiment.
(AVI)

Video S2 Movie of one simulation showing the motions of the moving particles (black dots) as well as the surface particles (red dots) which cover the free surface.
(AVI)
