# Correction: Collective Cell Motion in an Epithelial Sheet Can be Quantitatively Described by a Stochastic Interacting Particle Model

**DOI:** 10.1371/journal.pcbi.1003717

**Published:** 2014-06-13

**Authors:** The PLOS Computational Biology Staff

The units of several parameters are incorrect. All the provided numerical values are correct with length measured in microns (*μm*) and time measured in hours (*h*). Specifically:

-in the caption of Figure 1, the third equation in the final sentence should read 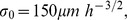



-in the captions of Figure 3 and Figure S5, the equation in the second sentence should read 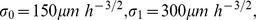



-in the Methods section, subsection "Details of the model", the first equation in the text after Eq. (4) should read 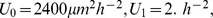



-in the Methods section, subsection "Epithelium border, leader cells and cell interaction with free surface", at the end of the sentence containing Eq. (6) and in the subsequent sentence, the units of *A_s_* are missing. *A_s_* is given in 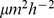.
