# Supplementary figures and images for: Collective Cell Motion in an Epithelial Sheet Can Be Quantitatively Described by a Stochastic Interacting Particle Model
Source: PLoS Comput Biol. 2013 Mar 7;9(3):e1002944. doi: 10.1371/journal.pcbi.1002944 (PMC3591275; doi:10.1371/journal.pcbi.1002944)

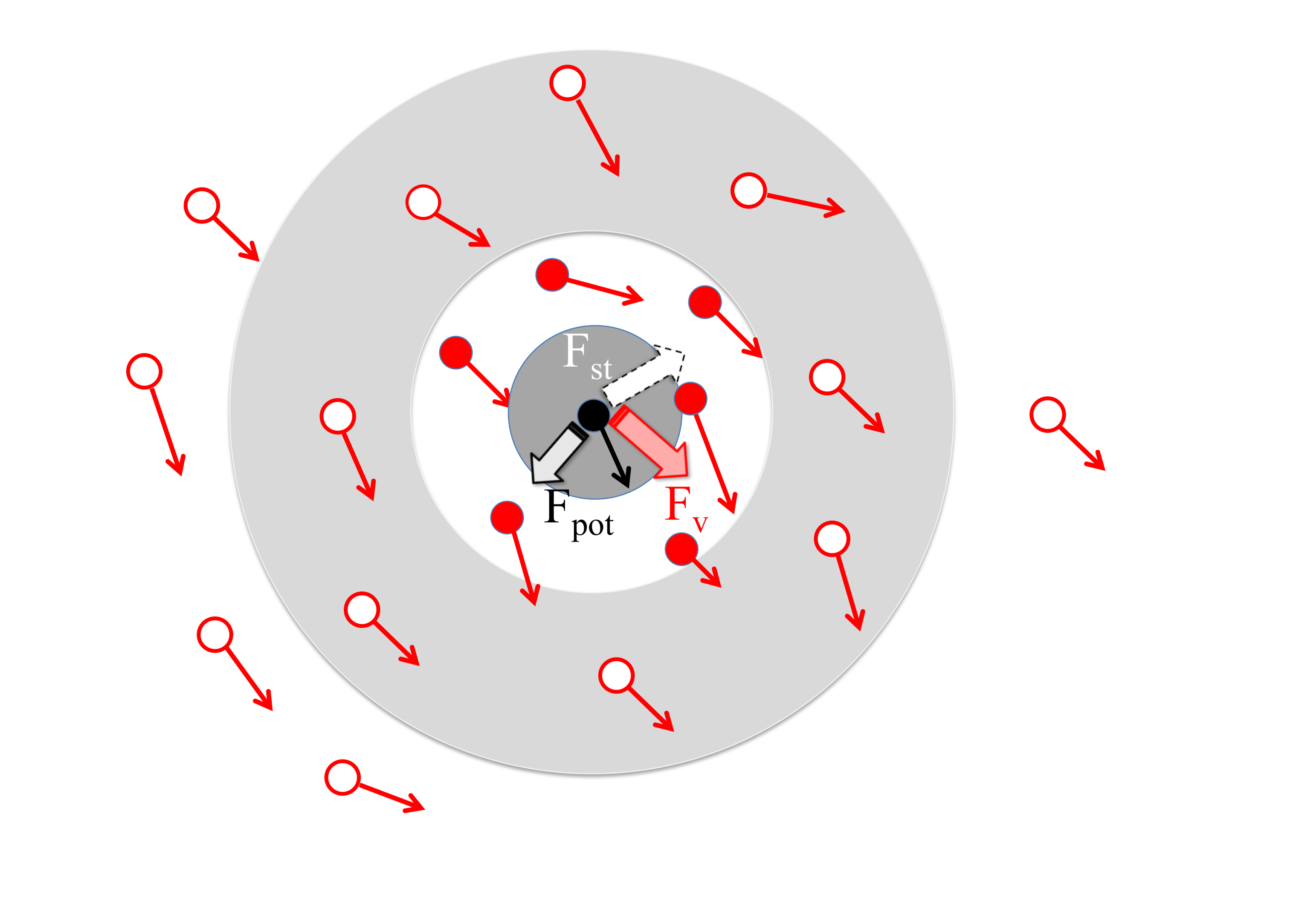

Supplement: Figure S1 — Sketch of the model. In the model each particle (here depicted by a small disk or a circle) has its own velocity (thin solid arrow). A given particle (denoted here by a solid black circle) interacts with its nearest neighbors (solid red disks). It moves under the action of three forces (large open arrows) which modify the particle velocity: one stochastic force and two interaction forces, an alignment force between velocities and a force generated by a potential . The chosen potential is such that is repulsive at short distance (here represented by the dark grey disk), vanishes in an intermediate range of distances (white annulus) and is attractive in a still larger-range of distances (lighter annulus). Note that a particle interacts only with its nearest neighbors and not with particles that are farther from it (denoted here by open red circles). (TIF) [file pcbi.1002944.s001.tif]

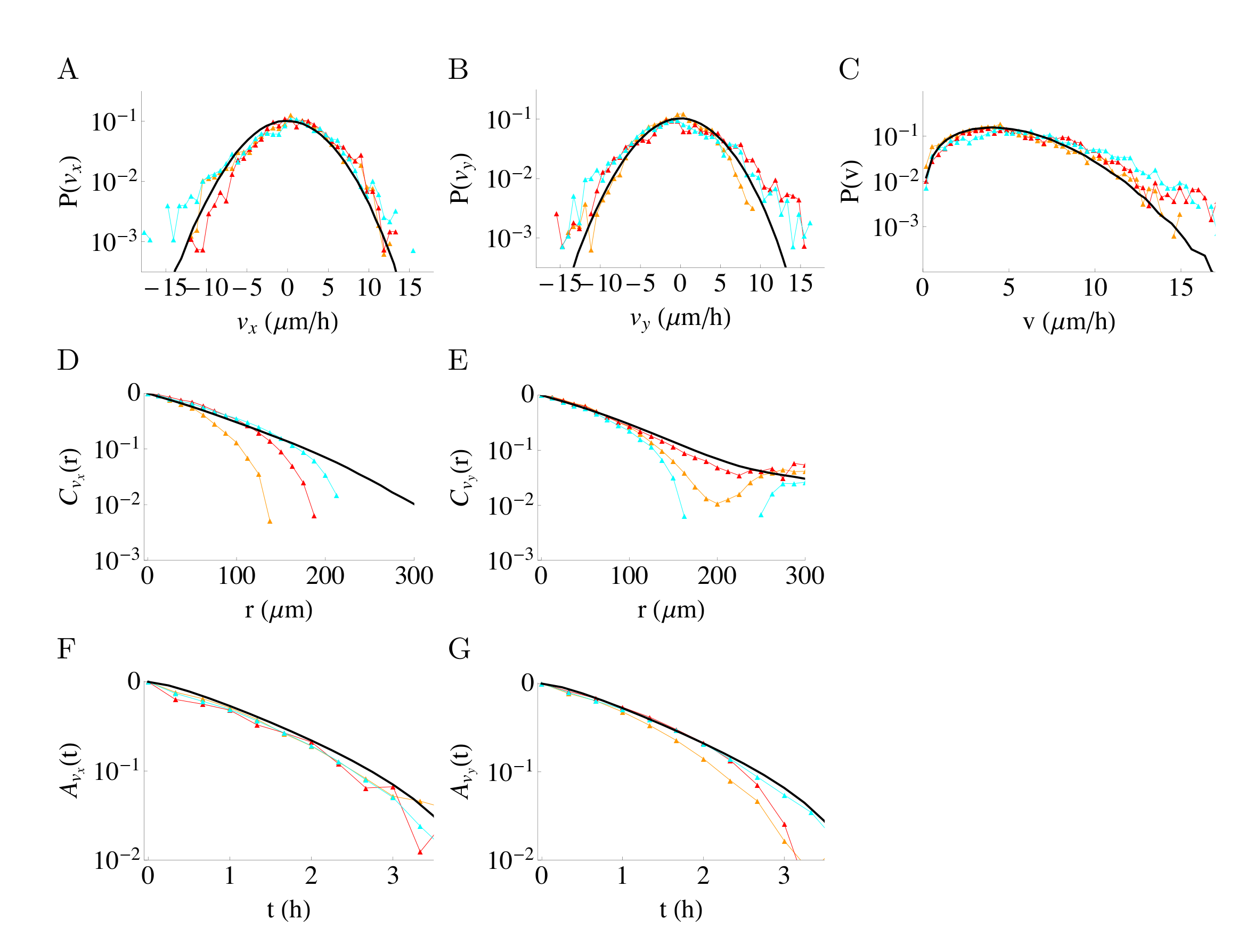

Supplement: Figure S2 — Statistical characterization of the cell velocity field, tails of the distribution functions. Data shown in Figure 2 of the main text are replotted in log-linear plots to better display the tails of the cell speed distribution functions and of the velocity-velocity correlations. The model results are depicted by solid black lines and the results of 3 experiments are depicted colored triangles and lines. (A, B, C) Probability distributions of the components of the velocity and of the velocity modulus. They are well-approximated respectively by Gaussian and Lorentzian distributions. (D,E) Equal time correlations of the velocity components as a function of distance. The experimental data show large relative fluctuations at long distances and the correlations become small. In this region, experimental data can take negative values and the corresponding colored lines are interrupted since the data cannot be plotted in log coordinate. (F,G) Temporal auto-correlations of the velocity components are close to exponential both in the model and in the experiments. (TIF) [file pcbi.1002944.s002.tif]

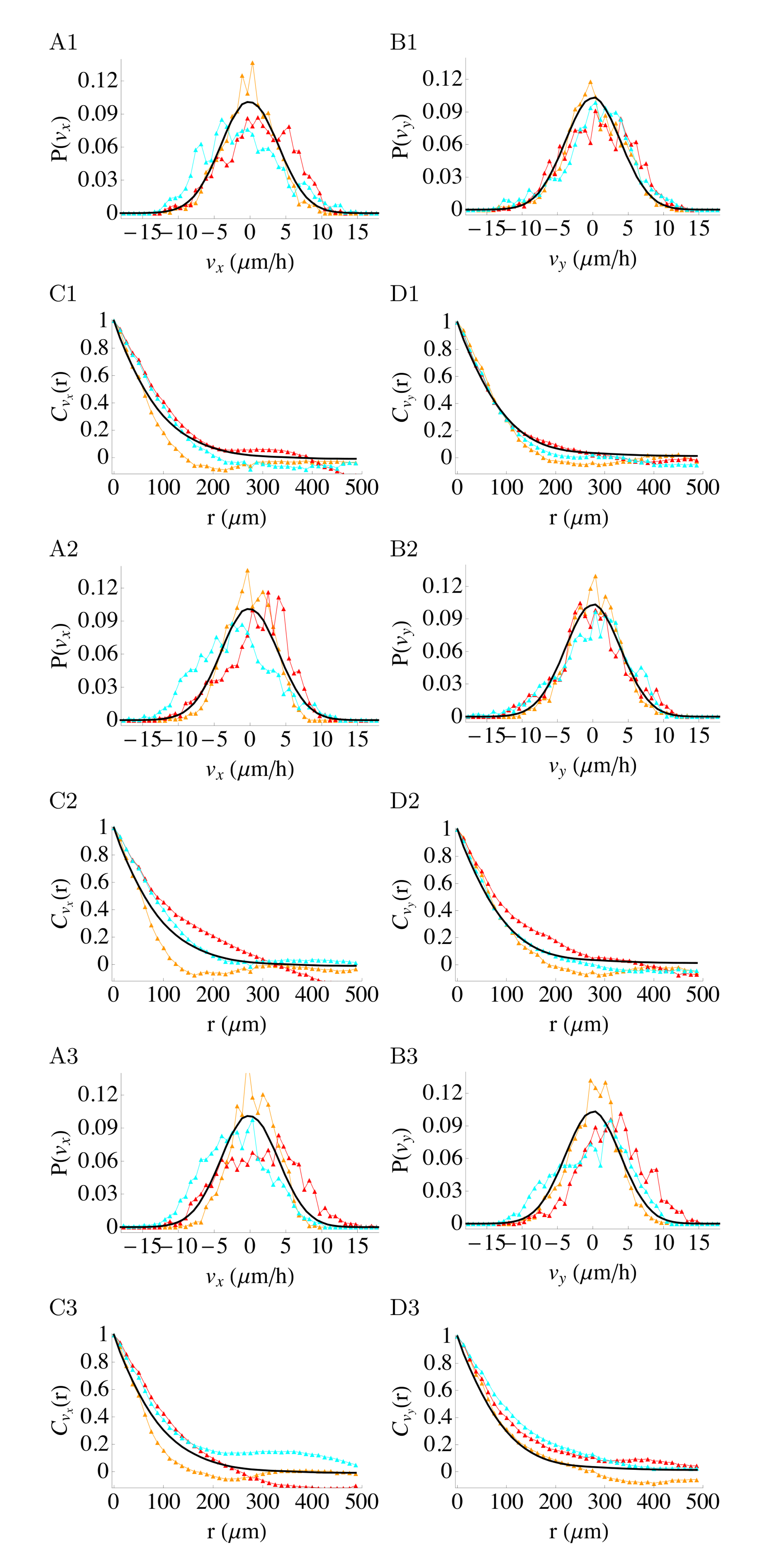

Supplement: Figure S3 — Statistical characterization of the cell velocity as in Figure 2 of the main text but at later times after stencil removal: 1 h (A1, B1, C1, D1), 2 h (A2, B2, C2, D2) and 3 h (A3,B3,C3,D3). As in Figure 2, experimental data (colored symbols) are taken from the motions of cell in a center square of in 3 experiments with initial bands of cells of size . The solid lines show the corresponding model fits 30 min after stencil removal as in Figure 2. Panels (A) and (B) display and velocity component distributions. Panels (C) and (D) show correlations of and velocity components as a function of cell distances.The distributions and correlations remain in good agreement with the model fit. As time passes, the distributions and correlations depart from the early fits and from the corresponding functions since border motion starts to influence cell motion in the center of the band. (TIF) [file pcbi.1002944.s003.tif]

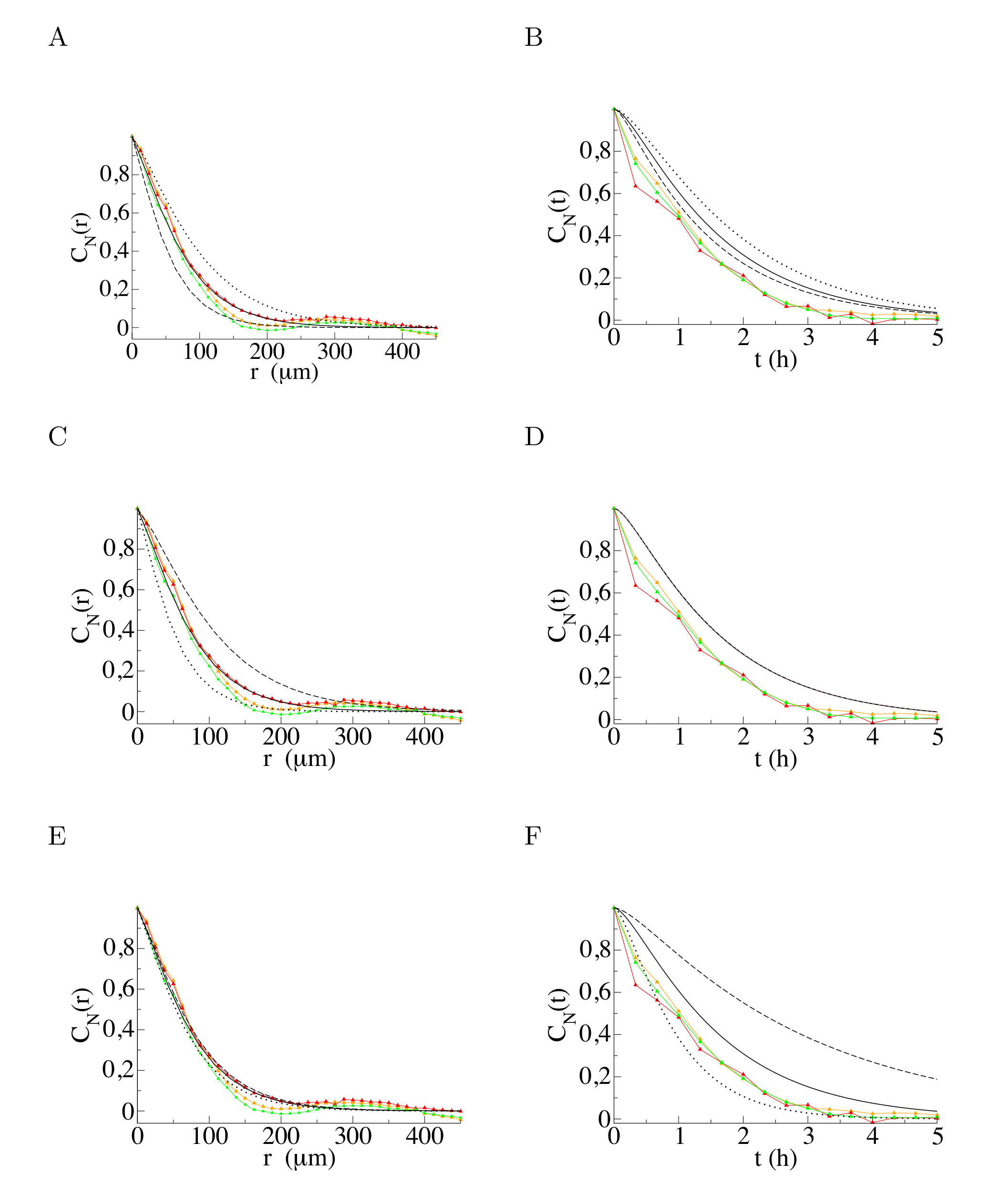

Supplement: Figure S4 — Analytical approximations of correlation functions with cell centers fixed on a triangular lattice showing the dependence of the correlation functions on different parameters. Panels (A),(C),(E) show the normalized spatial velocity correlations as a function of distance and Panels (B),(D),(F) the normalized velocity autocorrelations as a function of time. The parameter is varied in (A) and (B). The parameter is varied in (C) and (D). The parameter is varied in (E) and (F). The solid black lines are drawn for the values of given in Figure 1 of the main manuscript, the dashed black lines for a two times larger value of the varied parameter and the dotted lines for a half as large value. Experimental data are shown by colored symbols for reference. One can note in (B),(D),(F) that the time velocity autocorrelation decays more slowly in the approximation that in the model with moving cells (compare with Figure 2 I in the main text). (TIF) [file pcbi.1002944.s004.tif]

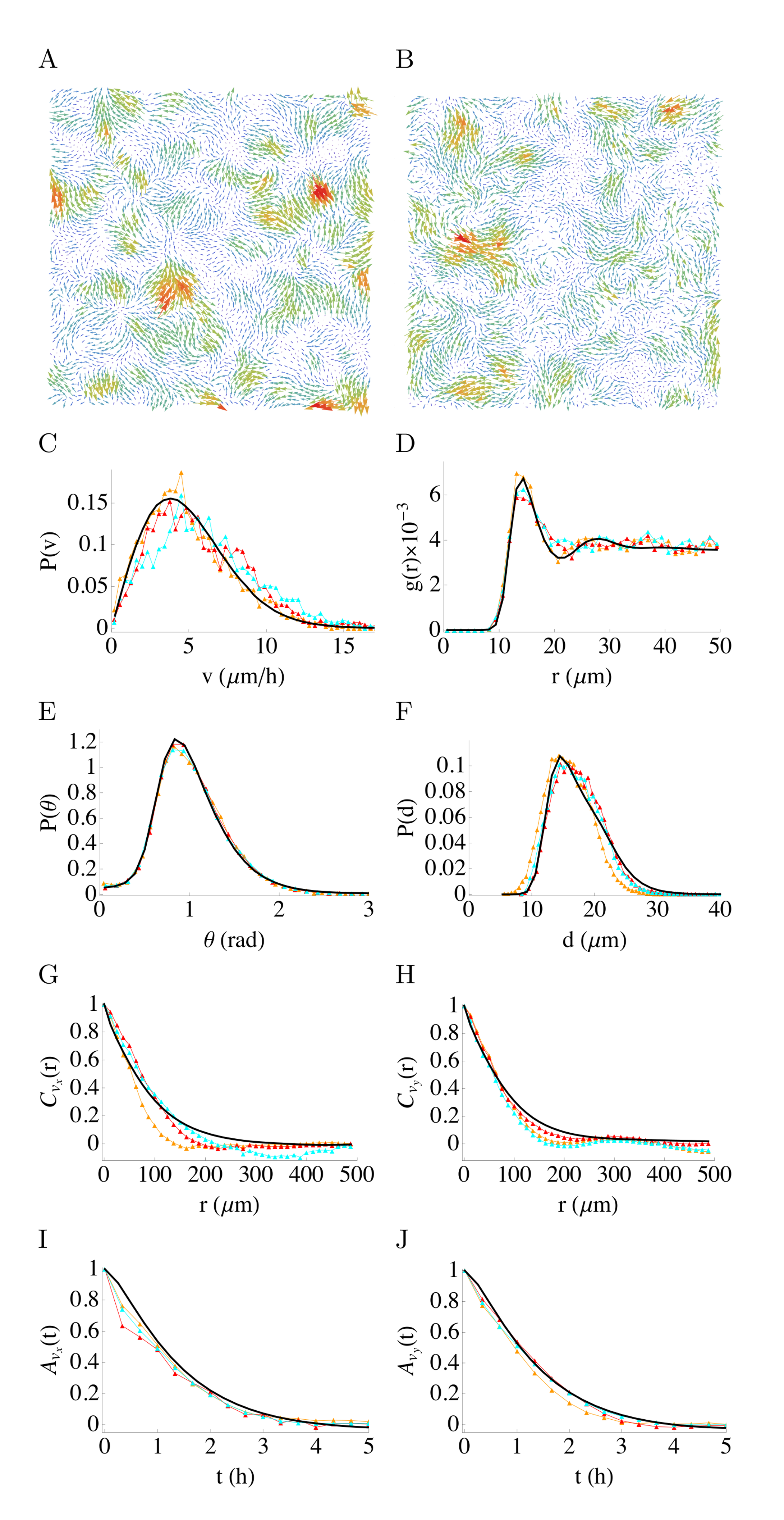

Supplement: Figure S5 — Statistical characterizations of the cell velocity field and positions at early time (30 min after stencil removal). Same as Figure 1 and Figure 2 of the main text for a model with noise amplitude varying with density see Methods ). Other parameters are the same as in Figure 1 and Figure 2 of the main text. Results of numerical simulations of the model are shown as solid black curves. The experimental data of 3 different experiments are shown as colored symbols for comparison. (A) Example of velocity field in the experiment and (B) in the simulation. (C) Distribution of the cell velocity moduli, (D) spatial correlation of the cell centers, (E) distribution of the angle between two successive neighbors of a cell (see text), (F) distribution of the distance between the centers of two neighboring cells, (G) and (H) spatial velocity correlation for the components and of the velocity respectively, (I) and (J) temporal velocity correlation for the components and of the velocity respectively. (TIF) [file pcbi.1002944.s005.tif]

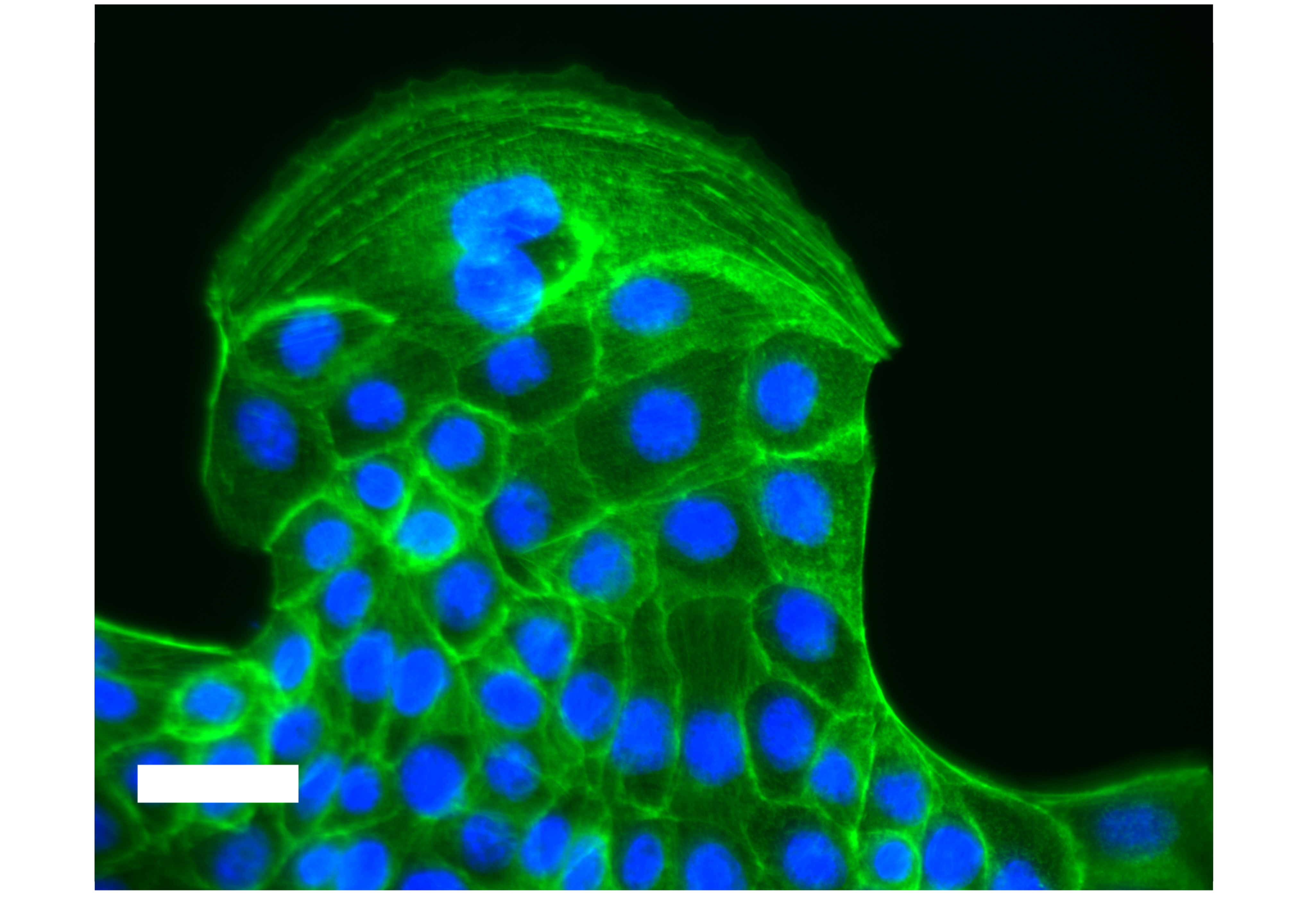

Supplement: Figure S6 — Picture of a leader cell and a finger. Cells were fixed and stained for nuclei with DAPI (blue) and for F-actin with alexa488-conjugated phalloidin (bar: ). It is clearly seen that the leader cell is bi-nucleated and that it displays a well-developed lamellipodium. (TIF) [file pcbi.1002944.s006.tif]

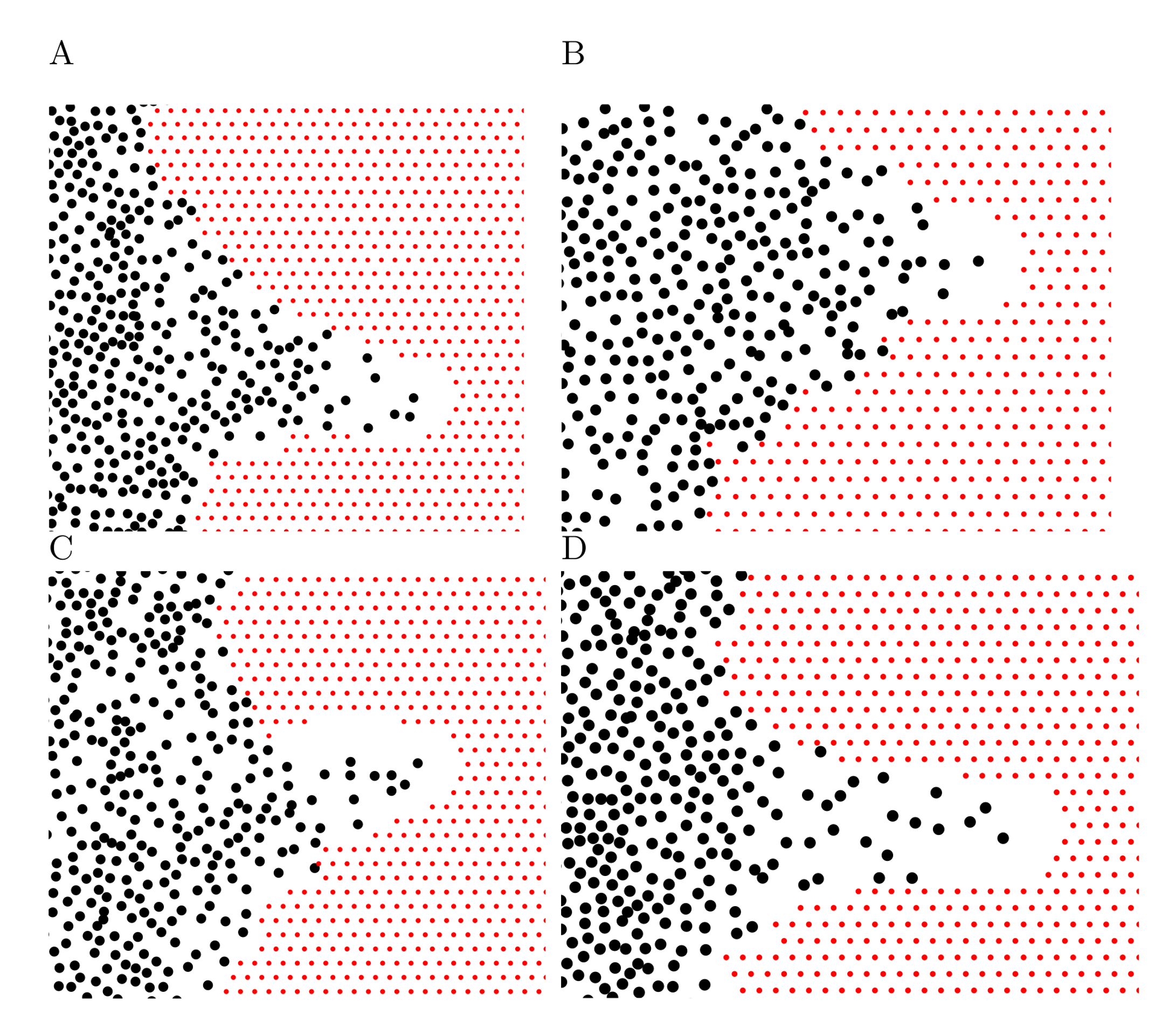

Supplement: Figure S7 — (A, B, C, D) Some simulated fingers with the free-surface repellent particles shown in red. The free-surface particles were used to draw the solid line which was considered as the finger contour. This contour was used to measure the finger density as a function of position, as shown in Figure 3 D of the manuscript. (TIF) [file pcbi.1002944.s007.tif]

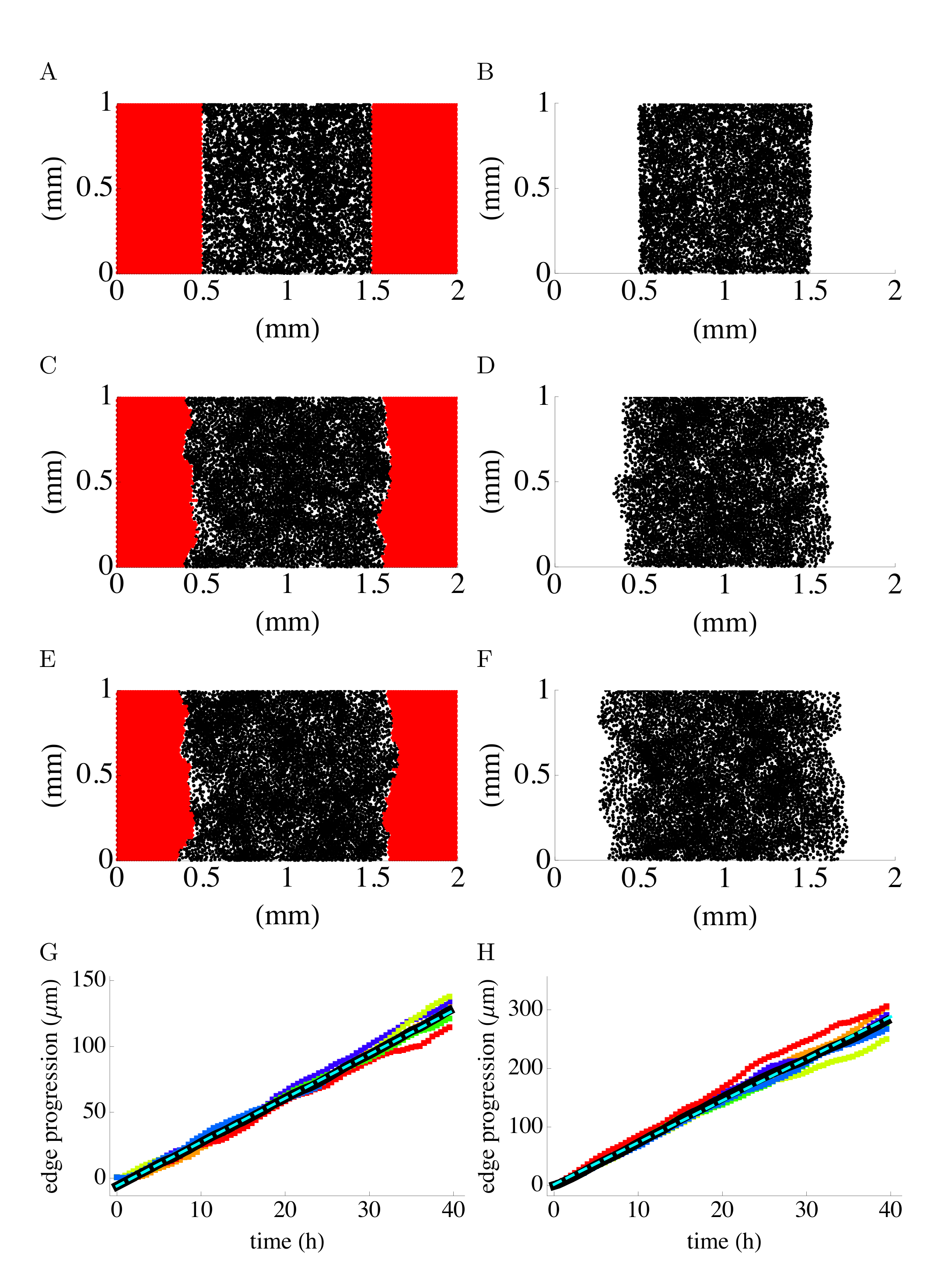

Supplement: Figure S8 — Border progression without the addition of leader cells and restrained by free -surface repulsion (left column A,C,E,G) or “unrestrained” i. e. without surface repulsion (right column, B,D,F,H). Images of two simulations at different times after free-surface unmasking (A & B, ; C & D, ; E & F, ). G & H: Border progression in () simulations (colored symbols) together with their average (solid black line) and linear fits (dashed light blue line which give a mean border progression speed of (G) in the restrained case and of (H) in the unrestrained case. (TIF) [file pcbi.1002944.s008.tif]

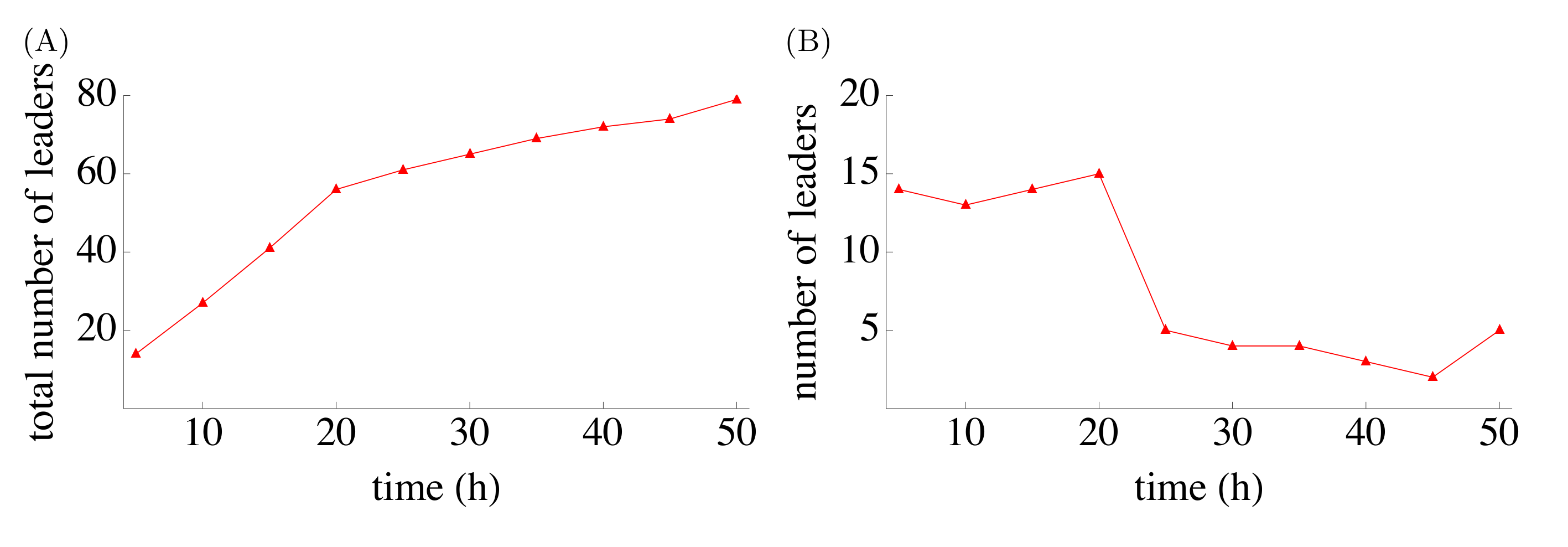

Supplement: Figure S9 — Experimental data on leader appearance along the epithelium border. (A) Total number of leader cells as a function of time in different experiments corresponding to a cumulated epithelium border length of 17 mm. (B) Same data showing the number of leader cells appearing during different time intervals (time bin : 5 h). (TIF) [file pcbi.1002944.s009.tif]

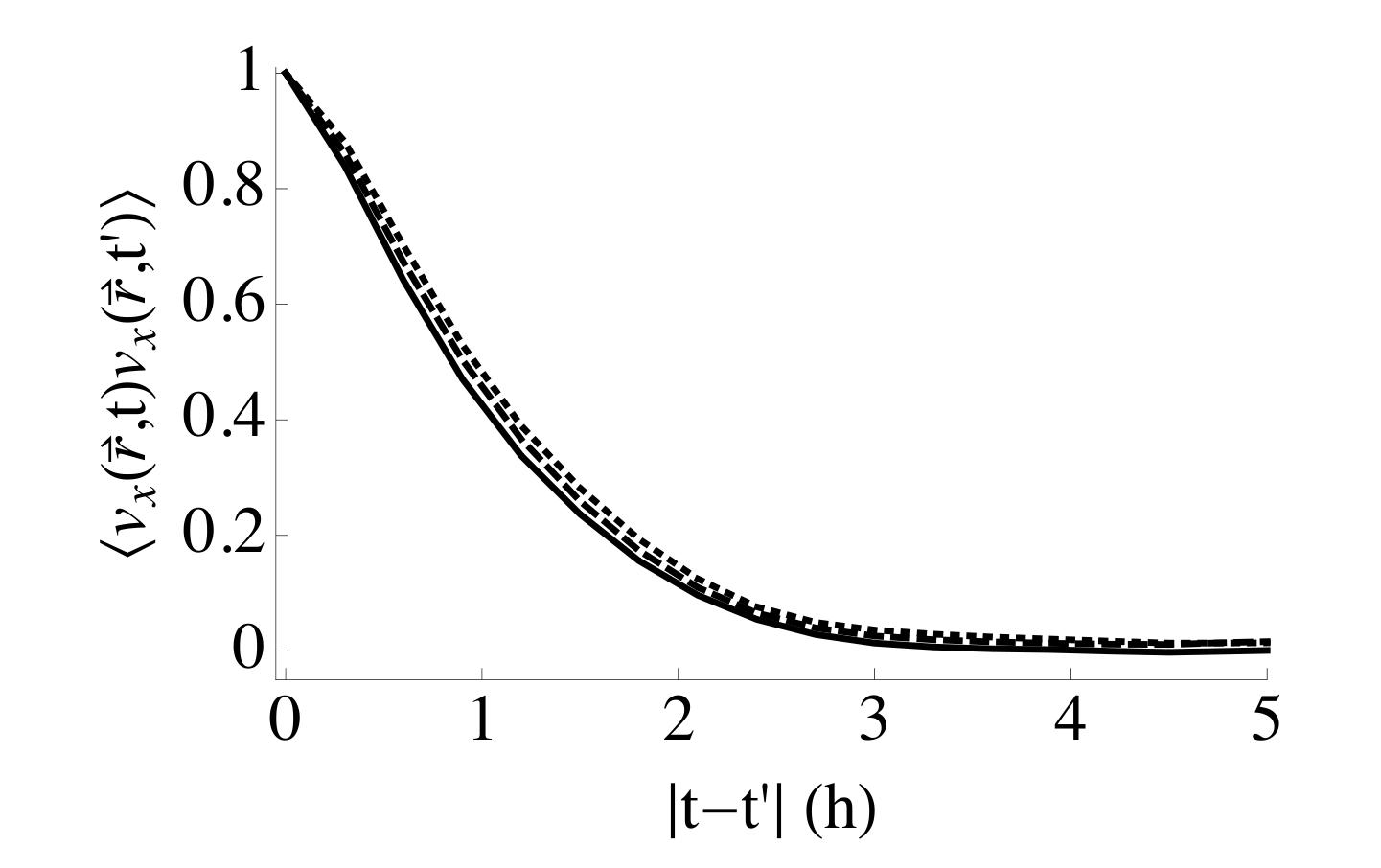

Supplement: Figure S10 — Comparison of the velocity auto-correlation function for the particles and for the velocity field in the model with constant noise amplitude. The cell velocity autocorrelation is shown as a continuous curve. The velocity field was defined by attributing to the center of each square of a square-grid the mean velocity of the particles that it contained. Two different squares grids were used of step size and (dashed and dotted curves). The corresponding auto-correlation are shown as dashed () and dotted () curves. The different curves are very close. To gauge the difference in parameter estimates, the model velocity field auto-correlations were used to fit the PIV velocity field. The obtained values of the parameters did not significantly differ from those used in Figure 1 and Figure 2 (the obtained were (1.43, 38, 1.40) for the continuous curve, (1.42, 38,1.41) for the dashed curve; and (1.42, 38, 1.40) for the dotted curve). (TIF) [file pcbi.1002944.s010.tif]

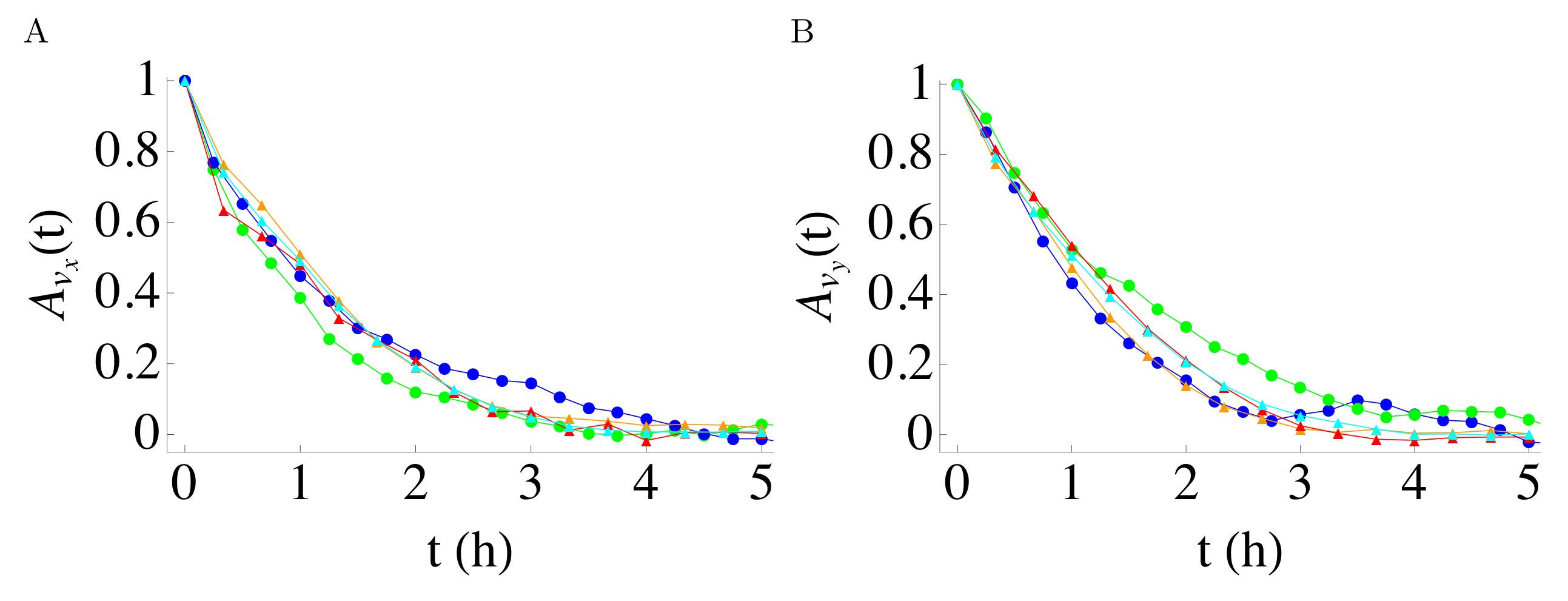

Supplement: Figure S11 — Comparison of the cell velocity auto-correlation functions (circles, experiments) to velocity field auto correlation (triangles; experiments) in the experiments. (A) Autocorrelation of the velocity component. (B) autocorrelation of the velocity component. (TIF) [file pcbi.1002944.s011.tif]
