# Supplementary material for: Collective Cell Motion in an Epithelial Sheet Can Be Quantitatively Described by a Stochastic Interacting Particle Model
Source: PLoS Comput Biol. 2013 Mar 7;9(3):e1002944. doi: 10.1371/journal.pcbi.1002944 (PMC3591275; doi:10.1371/journal.pcbi.1002944)
Supplement: Text S1 — Velocity correlation functions in an analytically solvable approximation of the model and some simple estimates on border progression resulting from leader cell creation. (PDF) [file pcbi.1002944.s012.pdf]

Text S1: Collective cell motion in an epithelial sheet can be quantitatively described by a stochastic interacting particle model

Néstor Sepúlveda, Laurence Petitjean, Olivier Cochet , Erwan Grasland-Mongrain, Pascal Silberzan, and Vincent Hakim

I. VELOCITY CORRELATION FUNCTIONS IN AN ANALYTICALLY SOLVABLE APPROXIMATION OF THE MODEL.

The model formulated and simulated in the main text is difficult to solve analytically. We consider in the present appendix an analytically solvable approximation of the model that eases parameter exploration and the analysis of the role of the different parameters. We keep the velocity and noise equations (Eq. [1,2]) of the main text but consider the positions of the particles fixed at the vertices of a regular lattice. With this approximation, the repulsive inter-particle forces cancel each other in Eq. [1]. As a consequence, the velocity equations become linear, and therefore solvable. The cell velocity equations thus reduce to

$$\frac{dv^\mu_{\mathbf{j}}}{dt} = -\alpha\, v^\mu_{\mathbf{j}} + \frac{\beta}{N_{\mathcal{V}}} \sum_{\mathbf{k}\in\mathcal{V}_{\mathbf{j}}} (v^\mu_{\mathbf{k}} - v^\mu_{\mathbf{j}}) + \sigma\, \eta^\mu_{\mathbf{j}}, \tag{1}$$

$$\tau \frac{d\eta^\mu_{\mathbf{j}}}{dt} = -\eta^\mu_{\mathbf{j}} + \xi^\mu_{\mathbf{j}}, \tag{2}$$

where the velocity are assigned to the vertices $\mathbf{j}$ of a regular lattice. The index $\mu = 1, 2$ denotes the two components, along x and y, of the two-dimensional vectors, $\mathcal{V}_{\mathbf{j}}$ the set of the nearest neighbors of lattice point $\mathbf{j}$, $N_{\mathcal{V}}$ the cardinal of $\mathcal{V}_{\mathbf{j}}$ (i.e. the number of these neighbors) and $\xi^\mu_{\mathbf{j}}$ a white-noise field

$$\langle \xi^\mu_{\mathbf{j}}(t)\, \xi^{\mu'}_{\mathbf{j}'}(t')\rangle = \delta(t-t')\, \delta_{\mathbf{j},\mathbf{j}'}\, \delta_{\mu,\mu'}. \tag{3}$$

The velocity field is a gaussian field since it is the linear transform of the white-noise gaussian field ξ. As such, it is completely characterized by its two-point correlation function which we compute below. In the following, we focus on the triangular lattice where each cell has six neighbors and, for comparison, we give also the corresponding expressions for the square lattice where each cell has four neighbors . Both lattices can be written as the set of points with integer coordinates on two basis vectors $\mathbf{u}_1$, $\mathbf{u}_2$

$$\mathbf{j} = j_1\mathbf{u}_1 + j_2\mathbf{u}_2, \quad j_1 \in \mathbb{Z}, \quad j_2 \in \mathbb{Z}, \tag{4}$$

with

$$\text{for the square lattice : } \mathbf{u}_1 = a_S\, \mathbf{e}_x,\ \mathbf{u}_2 = a_S\, \mathbf{e}_x, \tag{5}$$

$$\text{and the triangular lattice : } \mathbf{u}_1 = \frac{\sqrt{3}}{2} a_T\, \mathbf{e}_x + \frac{1}{2} a_T\, \mathbf{e}_y,\ \mathbf{u}_2 = a_T\ \mathbf{e}_y, \tag{6}$$

where vector $\mathbf{e}_x$ and $\mathbf{e}_y$ are two orthonormal vectors in the plane and a_S, a_T the internode distances of the square and triangular lattice respectively. The internode distances of the square and triangular lattices are related to the density ρ of lattice points, i. e. to the cell number density, by

$$a_S^2 = \frac{1}{\rho}, \quad {a_T}^2 = \frac{2}{\sqrt{3}\,\rho}. \tag{7}$$

Eq. (1,3) are easily solved using Fourier transforms with, for instance, for the noise

$$\tilde{\xi}^\mu_{\mathbf{q}} = \sum_{\mathbf{k}} \exp(-i\mathbf{q}\cdot\mathbf{k})\, \xi^\mu_{\mathbf{k}}, \tag{8}$$

and similar formulas for the other fields. To this end, it is convenient to introduce the vectors $\mathbf{q}_1, \mathbf{q}_2$ of the reciprocal lattices such that

$$\mathbf{q}_n \cdot \mathbf{u}_m = \delta_{m,n},\ m = 1, 2,\ n = 1, 2. \tag{9}$$

For the square lattice, one has simply

$$\mathbf{q}_1 = \frac{1}{a_S}\mathbf{e}_x, \mathbf{q}_2 = \frac{1}{a_S}\mathbf{e}_y, \tag{10}$$

while for the triangular lattice, $\mathbf{q}_1$ and $\mathbf{q}_2$ read

$$\mathbf{q}_1 = \frac{2}{\sqrt{3}\,a_T}\mathbf{e}_x, \mathbf{q}_2 = -\frac{1}{\sqrt{3}\,a_T}\mathbf{e}_x + \frac{1}{a_T}\mathbf{e}_y. \tag{11}$$

The velocity field can then be represented as

$$v_{\mathbf{j}}^{\mu}(t) = \sigma \int D^2\mathbf{q} \exp(i\mathbf{q}\cdot\mathbf{j}) \int_{-\infty}^{t} dt_1 \frac{\exp[\gamma(\mathbf{q})\,(t_1 - t)] - \exp[(t_1 - t)/\tau]}{1 - \tau\gamma(\mathbf{q})}\, \tilde{\xi}_{\mathbf{q}}^{\mu}(t_1), \tag{12}$$

where, with the wavevector $\mathbf{q}$ written as $\mathbf{q} = \rho_1\mathbf{q}_1 + \rho_2\mathbf{q}_2$, the integration $D^2\mathbf{q}$ is defined as the integration over ρ_1 and ρ_2 in the square domain $-\pi \leq \rho_1, \rho_2 \leq \pi$, $D^2\mathbf{q} \equiv d\rho_1 d\rho_2/(4\pi^2)$. Finally the function $\gamma(\mathbf{q})$ is defined by

$$\gamma(\mathbf{q}) = \alpha + \frac{\beta}{N_{\mathcal{V}}} \sum_{\mathbf{k}\in\mathcal{V}_{\mathbf{j}}} \{1 - \exp[i\mathbf{q}\cdot(\mathbf{k}-\mathbf{j})]\}. \tag{13}$$

More explicitly, one finds

$$\gamma_S(\mathbf{q}) = \alpha_S + \frac{\beta_S}{2}[2 - \cos(\rho_1) - \cos(\rho_2)], \tag{14}$$

$$\gamma_T(\mathbf{q}) = \alpha_T + \frac{\beta_T}{3}[3 - \cos(\rho_1) - \cos(\rho_2) - \cos(\rho_1 - \rho_2)], \tag{15}$$

for, respectively, the square and triangular lattice. We have added the index of the lattice on the constant β since it is interesting to compare the results for the two lattices with different values of β.

The velocity correlations are obtained by averaging the explicit representation of Eq. (12) over the white noise ξ using Eq. (8) and (3). This gives

$$\langle v_{\mathbf{j}}^{\mu}(t) v_{\mathbf{k}}^{\mu'}(t')\rangle = \sigma^2\delta_{\mu,\mu'} \int D^2\mathbf{q} \frac{\exp[i[\mathbf{q}\cdot(\mathbf{j}-\mathbf{k})]}{2[1-\tau^2\gamma^2(\mathbf{q})]} \left\{\frac{1}{\gamma(\mathbf{q})}\exp(-\gamma(\mathbf{q}) \mid t - t' \mid) - \tau\exp(\frac{-\mid t - t' \mid}{\tau})\right\}. \tag{16}$$

It should noted that the shapes of the correlation functions are independent of the noise amplitude which appears only as an overall multiplicative factor. The spatial and temporal correlation functions, as given by Eq. (16) and normalized to 1 at the origin, are plotted in Fig. S4.

II. LEADER CREATION AND BORDER PROGRESSION : SOME SIMPLE ESTIMATES

We consider a simple model in which a portion of interface without leader cells move at a slow speed v_s whereas a leader cell i appearing at $t_c(i)$ advances at a fast speed $v_f(i)$ at the tip of a finger of width $w(i)$.

At time t, the leader cell i stands at $x_i(t)$ with

$$x_i(t) = v_f(i)(t - t_c(i)) + v_s t_c(i). \tag{17}$$

For a uniform rate of creation of leader cells in time, the average time of creation of leader cells that appeared before time t is simply $t/2$. The average position of a finger tip at time t is thus

$$x_f(t) = \langle x_i(t)\rangle = (\overline{v}_f + v_s)t/2 \tag{18}$$

where we have denoted the mean leader cell speed by $\overline{v}_f$ and we have assumed that the speed of a leader cell is not correlated with the time of its appearance. The mean border position x_b in a given experiment is thus approximately given by

$$x_b(t) = \frac{1}{L}\left[\sum_i w_i x_i(t) + v_s t\,(L - \sum_i w_i)\right], \tag{19}$$

where L is the strip length (the initial rectilinear border length). Averaging over experiments, one obtains

$$\langle x_b(t)\rangle = \rho\, t\, \overline{w} x_f(t) + v_s t(1 - \rho \overline{w} t) = \rho(v_f - v_s)\overline{w}\, t^2/2 + v_s t, \tag{20}$$

where we have assumed that the total number of finger cells at time t is $\rho\, t L$. Taking a negligible $v_s = 3\mu$m/h, a rate of leader cell creation $\rho = 0.15$mm^{-1}h^{-1}, a mean leader cell velocity $\overline{v}_f = 18\,\mu$m/h and a mean finger width of 200μm gives $\langle x_b(t)\rangle \simeq .25\, t^2 \mu$m h^{-2} in reasonable agreement with experimental data and simulation results. If finger creation stops after $t = t_s$, then the epithelium border moves after t_c, at the velocity v_{as} it has reached at t_s namely

$$v_{as} = \rho(v_f - v_s)\overline{w}\, t_c + v_s. \tag{21}$$

Taking $t_c = 20 - 24$h, one obtains, $v_{as} = 7.5 - 8.5\,\mu$m/h again in reasonable agreement with the data.
